# Modifications in gene expression and phenolic compounds content by methyl jasmonate and fungal elicitors in *Ficus carica*. Cv. Siah hairy root cultures

**DOI:** 10.1186/s12870-024-05178-2

**Published:** 2024-06-10

**Authors:** Shahla Amani, Mehdi Mohebodini, Shahram Khademvatan, Morad Jafari, Vinod Kumar

**Affiliations:** 1 Department of Horticulture Sciences, University of Mohaghegh Ardabili, Ardabil, Iran; 2 Cellular and Molecular Research Center, Department of Medical Parasitology and Mycology, Urmia University of Medical Sciences, Urmia, Iran; 3 Department of Plant Production and Genetics, Urmia University, Urmia, Iran; 4 School of Water, Energy and Environment, Cranfield University, Cranfield, MK43 0AL UK

**Keywords:** Fungal elicitor, Hairy roots, Methyl jasmonate, *Piriformospora indica*, *Rhizobium (Agrobacterium) rhizogenes*, Secondary metabolites

## Abstract

**Background:**

One of the most effective strategies to increase phytochemicals production in plant cultures is elicitation. In the present study, we studied the effect of abiotic and biotic elicitors on the growth, key biosynthetic genes expression, antioxidant capacity, and phenolic compounds content in *Rhizobium (Agrobacterium) rhizogenes*-induced hairy roots cultures of *Ficus carica* cv. Siah.

**Methods:**

The elicitors included methyl jasmonate (MeJA) as abiotic elicitor, culture filtrate and cell extract of fungus *Piriformospora indica* as biotic elicitors were prepared to use. The cultures of *F. carica* hairy roots were exposed to elicitores at different time points. After elicitation treatments, hairy roots were collected, and evaluated for growth index, total phenolic (TPC) and flavonoids (TFC) content, antioxidant activity (2,2-diphenyl-1-picrylhydrazyl, DPPH and ferric ion reducing antioxidant power, FRAP assays), expression level of key phenolic/flavonoid biosynthesis genes, and high-performance liquid chromatography (HPLC) analysis of some main phenolic compounds in comparison to control.

**Results:**

Elicitation positively or negatively affected the growth, content of phenolic/flavonoid compounds and DPPH and FRAP antioxidant activities of hairy roots cultures in depending of elicitor concentration and exposure time. The maximum expression level of chalcone synthase (*CHS*: 55.1), flavonoid 3′-hydroxylase (*F3’H*: 34.33) genes and transcription factors MYB3 (32.22), Basic helix-loop-helix (bHLH: 45.73) was induced by MeJA elicitation, whereas the maximum expression level of phenylalanine ammonia-lyase (*PAL*: 26.72) and UDP-glucose flavonoid 3-O-glucosyltransferase (*UFGT*: 27.57) genes was obtained after *P. indica* culture filtrate elicitation. The *P. indica* elicitation also caused greatest increase in the content of gallic acid (5848 µg/g), caffeic acid (508.2 µg/g), rutin (43.5 µg/g), quercetin (341 µg/g), and apigenin (1167 µg/g) phenolic compounds.

**Conclusions:**

This study support that elicitation of *F. carica* cv. Siah hairy roots can be considered as an effective biotechnological method for improved phenolic/flavonoid compounds production, and of course this approach requires further research.

**Supplementary Information:**

The online version contains supplementary material available at 10.1186/s12870-024-05178-2.

## Introduction

*Ficus carica* (L.), known as fig tree, is the most important commercial fruit tree species of the large genus *Ficus* belonging to the Moraceae family [1]. As one of the oldest species domesticated, *F. carica* is native to eastern Mediterranean region and later spread to the western Mediterranean [1]. In different regions, fig cultivars are often distinguished based on the color, shape, and maturity of the fruit, local region or harvest date [2]. The peel color of *F. carica* fruits ranges from green to dark purple. Two important Iranian cultivars of *F. carica* are Siah as a fresh fig with dark purple peel color, and Sabz as dried fig with green peel color. The biological activities of antioxidant, antibacterial, anti-tumor, antiseptic, diuretic, anti-inflammatory, anti-helminthic, cancer prevention, etc. health properties have been reported from *F. carica* [3]. Traditionally, Leaves, fruits, and roots of *F. carica* have been used for the treatment of disorders such as respiratory disorders (coughs, sore throats, and bronchial problems), digestive disorders (loss of appetite, indigestion, diarrhea, and colic), dysentery, hemorrhoids, ulcers, cardiovascular disorders, diabetes, and skin disorders [4, 5]. It has been found that these biological activities of *F. carica* are derived from its phytochemicals, which are influenced by environmental and genetic factors and their interaction [6]. A high number of bioactive compounds such as phenolic acids, flavonoids, carotenoids, terpenoids, phytosterols, coumarins, furanocoumarins, vitamins, organic acids, fatty acid, and volatile compounds have been found in different parts of *F.carica* [5]. The gallic acid, chlorogenic acid, caffeic acid, ferulic acid, coumaric acid, syringic acid, and quinol, are the main phenolic acids and catechin, kaempferol, quercitin, rutin, and myricetin are the main flavonoids of *F. carica* [1, 4]. These phytochemicals (phenolic and flavonoid compounds) are synthesized through the phenylpropanoid and flavonoid pathway [7, 8].

In recent years, to enable industrial use, attention has been turned to the possibility of mass production of phytochemicals using natural sources [9]. Since the production rate of secondary metabolites is very low in nature, and may be negatively affected by unpredictable environmental/climatic fluctuations and disease, it is crucial to introduce efficient alternative strategies to increase the bioproduction of bioactive compounds [9]. Plant biotechnological techniques based on in vitro culture with advantages such as the ability of stable, predictable, efficient, and scalable secondary production, simple extraction and purification of phytochemicals, can be a reliable alternative for the rapid and uniform synthesis of bioactive compounds under controlled conditions [9, 10]. Plant hairy root culture (HRC) system, induced by *Rhizobium* (*Agrobacterium*) *rhizogenes*, has been recognized as in vitro stable platform for improvement of phytochemicals production [11]. Hairy roots can synthesize more than one metabolite and thus would be cost-effective for commercial production purposes [12]. Elicitation of hairy roots has been commonly used to stimulate the in vitro biosynthesis of secondary metabolites [12, 13]. Elicitation is an easy, high-efficiency, and low-cost technique that changes metabolic and biochemical pathways in hairy roots cultures by inducing oxidative stress and thereby activating the cellular immune and transcriptional responses for the secondary metabolism [14]. Elicitors are agents with abiotic (non-biological) and/or biotic (biological) origin [13]. Biotic elicitors include microorganism (fungi, bacteria, virus, etc.) and polysaccharide (chitin, pectin, cellulose, etc.) elicitors [13, 15]. The positive effects of endophytic fungi such as *Camarosporomyces flavigenus* [16], *Mucor fragilis* [17], *Chaetomium* sp. [18], on secondary metabolites production in plant tissue cultures has been reported. Abiotic elicitors include physical (light, drought, salinity, mechanical wounding, osmotic and thermal stresses), chemical (inorganic compounds and metals) and hormonal (jasmonates, salicylic acid, ethylene, etc.) elicitors [13, 15]. Jasmonates including, jasmonic acid (JA) and its derivative, methyl jasmonate (MeJA), have been introduced as potent elicitors in plant cultures in vitro due to their positive effects on promoting secondary metabolism [19]. For example, methyl jasmonate (MeJA) treatment increased production of asiaticoside in hairy root cultures of *Centella asiatica* [20], rosmarinic acid accumulation in hairy root cultures of *Prunella vulgaris* [21], phenolic acids accumulation in hairy root cultures of *Salvia miltiorrhiza* [22], and, anthraquinone production in hairy root cultures of *Rubia tinctorum* [23]. To better understand the effect of elicitors on the production of secondary metabolites of different plants, it is necessary to investigate the expression of biosynthetic pathway genes after elicitation.

In our previous study [24], hairy roots induction was optimized in *F. carica* (Siah, Sabz cultivars) and then the effects of MeJA on the contents of some phenolic compounds in hairy root cultures of both cultivars of *F. carica* were investigated, though the effects of MeJA and/or fungal elicitation on gene expression were not studied in Siah cultivar of *F. carica*. In this work, the effects of MeJA and fungal elicitation on the accumulation of phenolic/flavonoid compounds, antioxidant activity and expression levels of key genes involved in phenolic/flavonoid biosynthetic pathway have been evaluated in hairy roots cultures of *F. carica* cv. Siah.

## Materials and methods

### Chemicals reagents

All reagents, standard compounds, and chromatographic -grade solvents for biochemical analysis of phenolic acids (gallic acid, caffeic acid, chlorogenic acid, ρ-coumaric acid, rosmarinic acid, and cinnamic acid) and flavonoids (quercetin, rutin, and apigenin) were purchased from Merck (Darmstadt, Germany).

### Hairy roots cultures

**Fig. 1:**
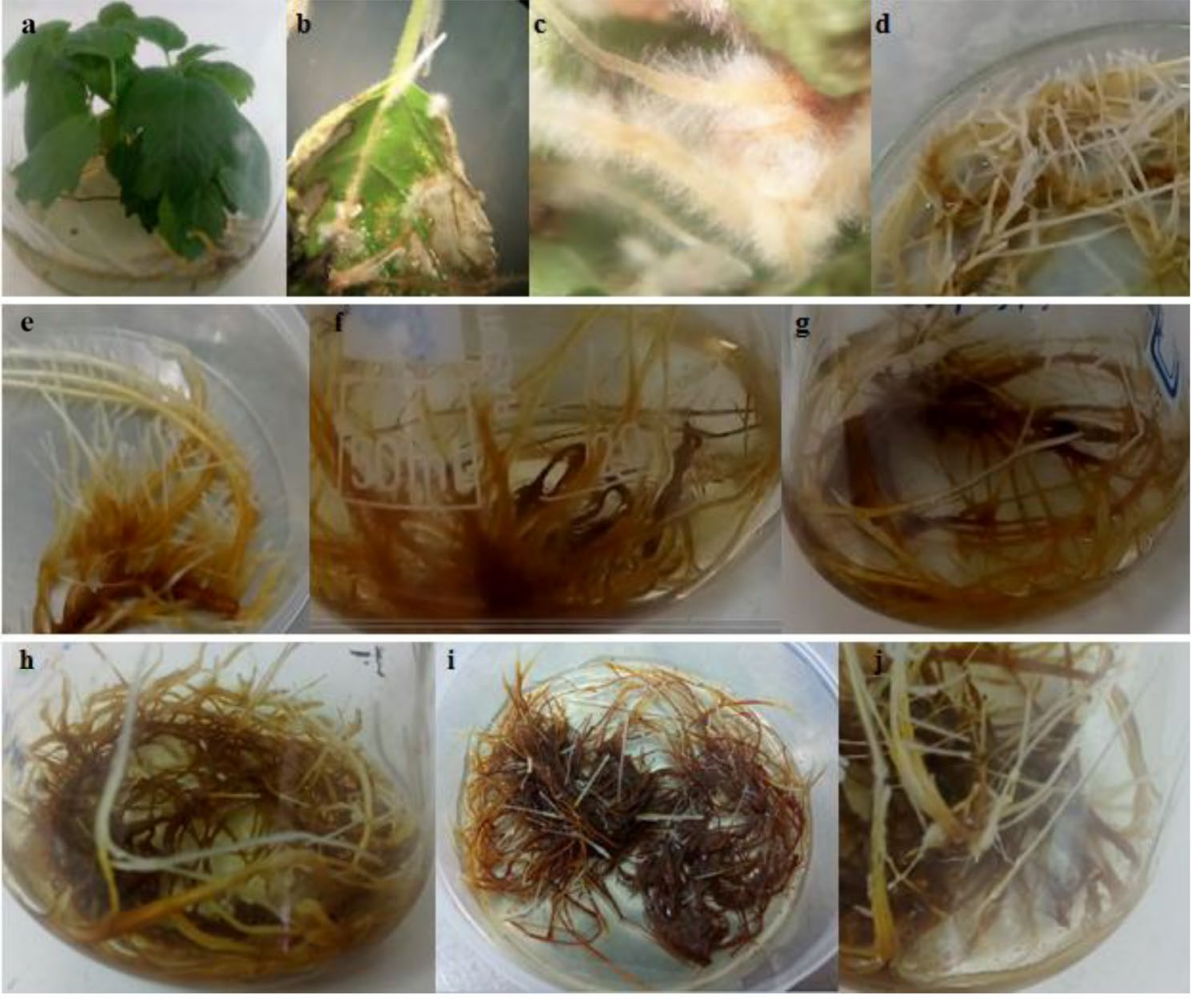
The in vitro culture of *F. carica* cv. Siah followed by induction, establishment and elicitation of hairy roots: *in vitro F. carica* cv. Siah plantlet (**a**); induced hairy root on the leaf of shoot explant (**b**); hairy root lines (**c**); highest growth hairy root line (L10) on solid medium culture (**d**); L10 hairy root line transferred to liquid culture (**e**); the growth of L10 hairy root line on liquid culture (**f** and **g**); L10 hairy root line before elicitation (**h**); L10 hairy root line after MeJA elicitation (**i**); L10 hairy root line after fungal elicitation (**j**)

In vitro seedlings of *F. carica* cv. Siah were cultured on woody plant medium, WPM [25] including vitamins and supplemented with 2% (w/v) sucrose, and 0.6% (w/v) agar and maintained at growth conditions of 25 ± 2 ºC with 16 h light/8 h photoperiod (Fig. 1, a). The hairy root induction was performed by agroinfection of shoot explants with *Agrobacterium rhizogenes* A7 [24]. Induced hairy roots were separated, numerical named as lines, transferred to hormone-free ½ Murashige and Skoog, MS solid medium + 300 mg/L cefotaxime, and incubated at 25 ± 2 ºC in darkness. About 6 to 7 sub-culturing of hairy roots in fresh medium but with a lower concentration of cefotaxime than before were done every 15 d until the complete removal of the remaining bacteria. During the establishment period, high growth rate hairy root lines were clearly defined. The highest growth rate hairy roots line (Fig. 1.) was selected as superior line and cultured in hormone-free ½ MS liquid media (300 mg/ 30 mL). For further biochemical and molecular investigations, the cultures were maintained on a rotary shaker at 120 rpm in the dark at a room temperature of 25 ± 2 ºC.

### PCR confirmation of hairy roots

The genomic DNA from hairy root lines and in vitro natural (normal) roots were extracted using cetyltrimethyl ammonium bromide (CTAB) buffer as described by Pirttila et al. [26]. To molecularly confirm the integration of bacteria *rol* genes in the hairy root lines genome, their genomic DNA was amplified by polymerase chain reaction (PCR) using specific primers. Specific primers for *rol* genes amplification were 1724 A-1724B (forward-GTGCTTTCGCATCTTGACAG, reverse-TCTCGCGAGAAGATGCAGAA for *rol*A-B), *rol*B (forward-ATGGATCCCAAATTGCTATTCCCCCACGA, reverse-TTAGGCTTCTTTCATTCGGTTTACTGCAGC) and 1724-C-1724D (forward-CTGTACCTCTACGTCGACT, reverse-TCAGTCGAGTGGGCTCCTTG for *rol*C-D). The *vir*D2 primer (forward-ATGCCCGATCGAGCTCAAGT, reverse-CCTGACCCAAACATCTCGGCTGCCCA), related to the virulence gene located on the vir region of the *Agrobacterium* plasmid (non-transmissible to the plant tissue), was used to ensure the absence of any bacterial contamination in the hairy root lines. Natural roots were defined as negative control and Ri-plasmid DNA extracted by alkaline lysis method [27] from bacteria as positive control. The PCR program of reactions (20 µL of the reaction final volume including 7.25 µL of sterile double distilled water, 10 µL of Super PCR Master Mix (Cat No: YT1553, Yekta Tejhiz Azma, Tehran, Iran), 1.25 µL of DNA sample and 0.75 µL of each reverse primer), was set as: one cycle at 94 ºC for 5 min, followed by 30 cycles at 94 ºC for 1 min, 60 ºC for rolB or 58 ºC for other primers for 60 s, 72 ºC for 10 min. The size of PCR amplified products (*rol*A-B: 1794 bp, *rol*B: 780 bp, *rol*C-D: 1105 bp, *vir*D2: 338 bp) was confirmed by running on 1.2% agarose gel electrophoresis stained with ethidium bromide.

### Elicitation

**Fig. 2:**
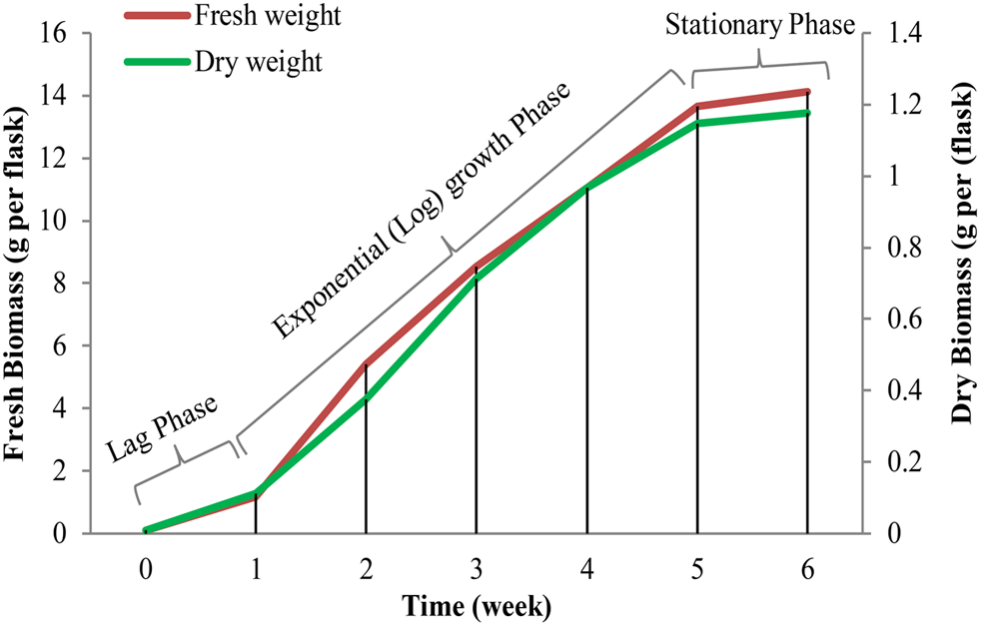
The growth curve of the superior hairy root line (L10) of *F. carica* cv. Siah. Lag phase: delayed growth phase, exponential (log) growth phase: logarithmic growth phase, stationary phase: plateau phase

The optimal hairy root culture age for elicitation varies by plant species. In the most cases, mid- to late-growth phase or the onset of stationary phase would be the appropriate time point to challenge cells for maximum response to elicitors [28, 29]. In this study, elicitation experiments were carried out on 30th days (late growth phase (Fig. 2.) [24]) of superior hairy root cultures. For this purpose, inocula of 500 mg fresh weight of the superior hairy roots cultures were placed in hormone-free ½ MS liquid media, and maintained for 30 d without subculture on rotary shaker under growth conditions (25 ± 2 ºC in darkness). After that, the culture medium of thirty-day-old cultures was removed, and after weighing the fresh weight of hairy roots, it was replaced with the fresh culture medium containing elicitors and incubated at the same conditions. Three elicitors including MeJA, *P. indica* culture filtrate, and *P. indica* cell extract were used. To prepare MeJA stock solution, the colorless viscous liquid of MeJA was dissolved in absolute methanol, sterilized by passing through a 0.22 micron sterile filter and stored at 4 ºC until use. To prepar fungal elicitors, *P. indica* fungus were cultured in Kafer’s liquid culture medium (Kafer, 1977). After 8 to 10 days of cultivation, the media of *P. indica* cultures was used to prepare the culture filtere elicitor and the *P. indica* biomass was used to prepare the cell extract elicitor. The collected media of *P. indica* cultures was passed through filter paper, 15 min centrifuged at 7500 rpm, and after filter sterilizing using a 0.22 micron Millipore syringe filter stored at 4 °C as culture filtrate elicitor until use. The fresh weighed biomass of *P. indica* after autoclave sterilization, drying on filter paper and washing three times with sterile distilled water, were homogenized as 10 mg of biomass in 1 ml distilled water under sterile conditions and stored at 4 °C as cell extract elicitor until use.

The cultures for experiments were: (I) control samples- untreated thirty-day-old hairy roots; treated with (II) 100 µM MeJA; (III) 200 µM MeJA; (IV) 300 µM MeJA; (V) 2.0% v/v *P. indica* culture filtrate; (VI) 4.0% v/v *P. indica* culture filtrate; (VII) 6.0% *P. indica* culture filtrate; (VIII) 2.0% v/v *P. indica* cell extract; (IX) 4.0% v/v *P. indica* cell extract; (X) 6.0% *P. indica* cell extract. For phytochemical and molecular analysis, the samples were collected at 48 h, 72 h, and 96 h (due to approaching the stationary phase of the cultures (day 35), the extending of elicitation time was stopped). To record the dry weight (DW), fresh hairy roots were exposed to 30 °C for four d and then to 60 °C for 1–2 d. The growth index ([dry weight after elicitation -weight at before elicitation]/ weight before elicitation) of samples was calculated.

### RNA extraction, cDNA synthesis and expression analysis of phenolic/flavonoid pathway genes by real-time PCR

**Fig. 3:**
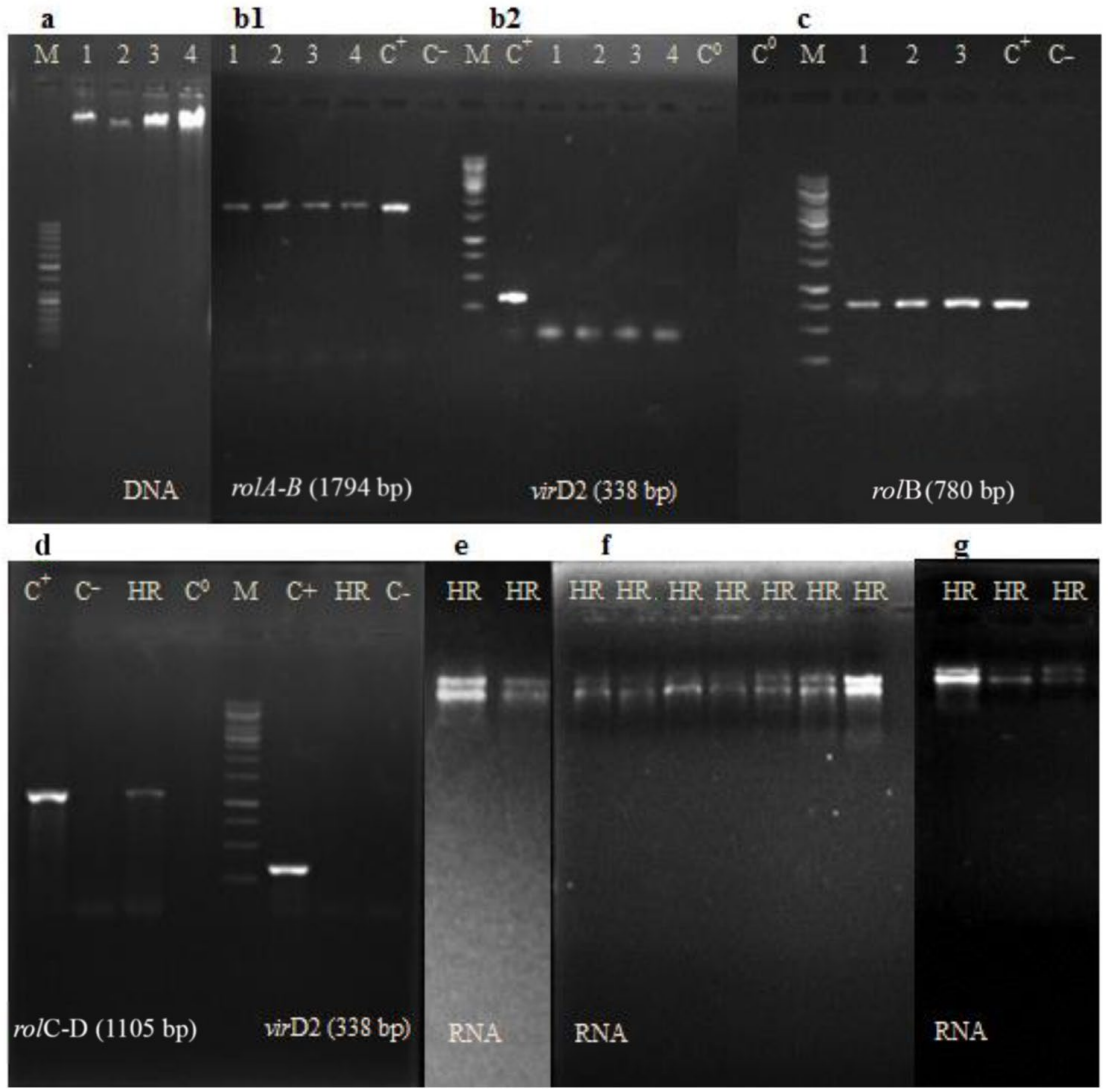
Gel electrophoresis of extracted DNA, polymerase chain reaction products amplified by rol and virD2 primers, and extracted RNA from hairy roots. **a**: DNA extracted from four hairy root lines; **b1**: PCR amplicons of four hairy root lines using rolA-B (1794 bp) primer and **b2**: confirmation of the absence of bacterial contamination in these four lines by PCR using virD2 (338 bp); **c**: PCR amplicons of three hairy root lines using rolB (780 bp); **d**: PCR amplicons of L10 hairy root line by rolC-D (1105 bp) and virD2; **e-g**: RNA extracted from non-elicited and/or elicited L10 hairy roots. M: DNA marker (50 bp or 1 kb) Ladder, GoldBio), 1–4: hairy root lines, C+: A7 *Agrobacterium rhizogenes* plasmid as positive control, C^_^: normal root as negative control, C^0^: PCR reaction without DNA as blank control, HR: L10 hairy root line (Additional edges and unrelated wells of gel have been cropped, from images, but the original image of all blots is available in the supplementary file.)

**Fig. 4:**
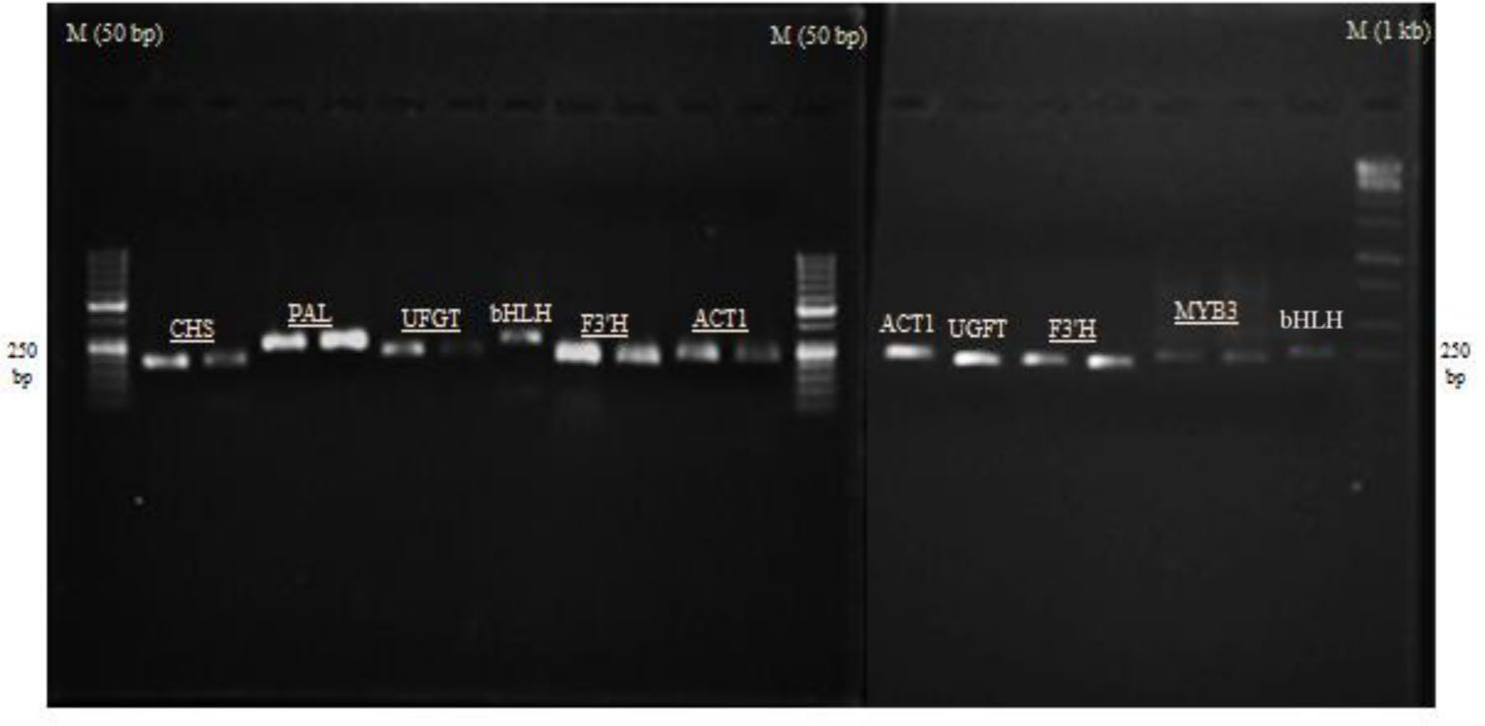
Gel electrophoresis of RTq-PCR amplicons using specific primers for *CHS*, *PAL*, *UFGT*, *F3’H*, *MYB3*, *bHLH*, and *ACT1* (as reference gene) genes. M: DNA marker (50 bp or 1 kb) Ladder (Additional edges and unrelated wells of gel have been cropped, from images, but the original image of all blots is available in the supplementary file.)

Total RNA extraction of elicited and control (non-elicited) hairy roots samples was performed using the CTAB based -RNA extraction protocol as optimized by Reid et al. [30]. Spectrophotometry using a NanoDrop 1000 (NanoDrop Technologies, Wilmington, USA) and electrophoresis in 1.2% agarose gel were assayed for quantitative and qualitative determination of each RNA sample (Fig. 3, e, f and g). First-strand cDNA synthesis was performed from 2 µl of each total RNA sample using the RevertAid First-Strand cDNA Synthesis Kit (Thermo Fisher Scientific, Lithuania) according to the kit instructions. The studied genes for the RTqPCR analysis were: phenylalanine ammonia-lyase (*PAL*), chalcone synthase (*CHS*), UDP glucose-flavonoid 3-O-glcosyl-transferase (*UFGT*), and flavanoid 3′-hydroxylase (*F3’H*) genes, and transcription factors of MYB3, and basic helix-loop-helix (bHLH). The specific primers pairs are detailed in Table 1. The RTqPCR assays were performed using the Rotor-Gene Q cycler (QIAGEN, USA) in a final volume of 20 µl including10 µl SYBER-Green Thermo Master mix (Thermo Fisher Scientific, Lithuania), 0.5 µl forward primer, 0.5 µl reverse primer, 2 µl cDNA sample and 7 µl of nuclease-free water. Amplification program was set as: 1 cycle of primary denaturing at 95 °C for 10 min followed by 40 cycles including secondary denaturing at 95 °C for 15 s, primer annealing at 54–60 °C for 40 s and extension at 72 °C for 45 s. The specificity of each primer pair by melting curve analysis and the size and quality of real time RT-PCR amplicons using 1.2% agarose gel electrophoresis were confirmed (Fig. 4). Relative quantitative analysis of gene expressions data was carried out by 2^−∆∆CT^ method with actin (ACT1) as reference gene.

**Table 1:** The specific primers for real time-quantitive PCR analysis

Primer Name		Sequence (5’ to 3’)	TA (˚C)	
ACT1 ACT1	Forward Reverse	GCTGGTCGTGATCTCACTGAC TCAGCACCGATTGTGATGACC	55	[51]
FcPAL FcPAL	Forward Reverse	GCAAGCCTTGAACTCTCCAC GGTTCTGCGAGAAGGATCTG	56	[52]
FcCHS FcCHS	Forward Reverse	CCGTGAAGTTGGGCFTTACAT AAACCACACTTGGCTTCCAC	54	[52]
FcUFGT2 FcUFGT2	Forward Reverse	CAGTGTCGTTTGCTGCAGAT AAGGAAGTCAACGGCGAGTA	60	[52]
FcF3′H1 FcF3′H1	Forward Reverse	GATCCGCCACCCTAAAATCT GGATGTGGTAGCCGTTGACT	54	[52]
FcMYB3 FcMYB3	Forward Reverse	GCAATTGCATTCAAGGGTTT GCCTTCCAGACACCAAATGT	54	[52]
FcbHLH FcbHLH	Forward Reverse	TACCACCACCACTCCTCCTC CCTCCTTGCCCTAACATGAA	56	[52]

### Extraction and quantification of phenolic and flavonoid compounds

#### Extraction

Dry tissue (100 mg) from elicited and non-elicited hairy roots cultures was powdered by using a mortar and pestle and three times extracted as follows: adding 3mL 80% MeOH, shaking for 5 h, 30 ºC water bath ultrasonicating for 20–60 min, 4500 rpm centrifuging for 15–20 min, collecting supernatant of each time and mixing for TPC, TFC and antioxidant assays. For HPLC analysis, the MeOH solvent of these extracts was evaporated and the dry extracts obtained were re-dissolved in 1.5 mL MeOH and after filtered through a 0.22 μm filter.

#### Total phenolic (TPC) and flavonoids (TFC) content assay

The TPC content (mg gallic acid equivalent (GAE)/g dry weight) of extracts was measured by using Folin-Ciocalteu reagent [31] and gallic acid as standard. The TFC content (mg quercetin (QE) equivalent/ g dry weight) of extracts was calculated via aluminum chloride (AlCl3) method [32] and quercetin as standard.

#### HPLC analysis of phenolic acids and flavonoids

The concentration of six phenolic acids including gallic acid, cinnamic acid, chlorogenic acid, rosmarinic acid, caffeic acid, coumaric acid, and three flavonoids including apigenin, quercetin and rutin in elicited hairy root cultures was determined by a HPLC system (Agilent 1100, USA) and compared with its content in control sample (non-elicited hairy root culture). The HPLC calibration curves were established using the absorption wavelength set at 272 nm for gallic acid, cinnamic acid and apigenin, 250 nm for chlorogenic acid, rosmarinic acid and quercetin, and in 310 nm for caffeic acid, coumaric acid and rutin (Supplementary Fig. 1). Determination of compounds was performed according method described in our previous study [24].

### Antioxidant analysis

Antioxidant assay of control and elicited hairy roots cultures was evaluated using colorimetric procedures of DPPH free radical scavenging according to [33] method and FRAP (ferric reducing antioxidant power) according to the method of [34]. The result of DPPH antioxidant activity was reported as percentage of scavenged DPPH radical (%). The result of FRAP antioxidant activity was expressed as the FeSO_4_.7H2O value (mol (M) FeSO_4_.7H_2_O /g dry weight of hairy roots) using FeSO_4_.7H_2_O as the standard.

### Statistical analysis

Analysis of variance (ANOVA) and Duncan’s multi-range test were performed using Statistical Package for the Social Sciences, SPSS version 22 software. Data were expressed as the mean of three replicates ± standard deviation (SD) and significance differences were indicated by lettering according to one-way ANOVA followed by Duncan’s test at 0.05 significance level.

## Results

### Establishment and confirmation of hairy roots

*A. rhizogenes*-transformed roots of *F. carica* cv. Siah were induced after 7 d of incubation from the wounded sites of explants infected with A7 strain (Fig. 1.). For the molecular confirmation of hairy root lines, the presence of rol genes was analyzed by PCR run using specific primers. Agarose gel electrophoresis (Fig. 3.) showed that the genes of *rol*A-B (1794 bp), *rol*B (780 bp), and *rol*C-D (1105 bp) were present in positive control (*A. rhizogenes* plasmid) and *A. rhizogenes*-transformed root lines but not in negative control (non-transformed natural roots). Amplification of 338 bp band of *virD2* gene was observed in only positive control sample (Fig. 3.). After PCR confirmation, hairy root line with the highest growth power was designated as the superior line (L10 line) and extensively subcultured for further analysis.

### Effect of elicitation on hairy roots growth

To show the effect of elicitation on hairy roots growth, the growth index of elicited hairy root cultures was determined and compared to control samples. As shown in Table 2, elicitation, depending on type, concentration and exposure time, had a significant effect on the growth index of hairy root cultures. MeJA elicitation dramatically decreased growth index of hairy roots samples in all treatments whereas, *P. indica* elicitation, except in high concentration treatments (6% culture filtrate and cell extract (v/v)), significantly increased it. In MeJA elicitation of hairy roots, with increasing the concentration of elicitor, the intensity of growth index reduction increased and with increasing exposure time, its intensity decreased. The highest increase in growth index of hairy roots was obtained after 48 h of treatment with 4% culture filtrate elicitor.

**Table 2:** *F. carica* cv. Siah hairy roots growth affected by MeJA and fungal elicitation

Elicitor	Concentration	Exposure time	ΔDW (mg)	GI (%)
MeJA (µM)	100	48	-165.29 ± 33.71^ij^	-16.12 ± 3.29^jk^
		72	-117.33 ± 37.38^hi^	-11.33 ± 3.68^ij^
		96	-388.02 ± 27.24^k^	-37.74 ± 2.63^n^
	200	48	-394.07 ± 8.07^k^	-44.72 ± 0.71^o^
		72	-207.26 ± 33.89^j^	-24.22± 3.56^lm^
		96	-110.67 ± 23.80^ghi^	-12.52 ± 2.51^ij^
	300	48	-559.10 ± 52.30^l^	-58.81 ± 4.20^p^
		72	-200.52 ± 41.67^j^	-30.37 ± 4.99^m^
		96	-157.52 ± 51.65^ij^	-20.20 ± 6.31^kl^
*P. indica* culture filtrate (% v/v)	2	48	143.77 ± 22.20^b^	20.81 ± 2.39^b^
		72	153.63 ± 7.20^b^	12.85 ± 0.55^c^
		96	171.98 ± 39.56^b^	25.38 ± 4.62^b^
	4	48	519.30 ± 33.20^a^	41.21 ± 2.82^a^
		72	78.94 ± 3.09^c^	22.84 ± 1.38^b^
		96	18.08 ± 7.49^de^	1.74 ± 0.58^ef^
	6	48	-428.54 ± 26.19^k^	-27.40 ± 2.38^m^
		72	-93.78 ± 28.18^gh^	-8.35 ± 2.49^ghi^
		96	-27.74 ± 3.02^ef^	-2.91 ± 0.22^fg^
*P. indica* cell extract (%v/v)	2	48	81.25 ± 2.84^c^	22.41 ± 0.92^b^
		72	11.65 ± 3.02^de^	1.90 ± 0.52^ef^
		96	28.66 ± 1.03^cde^	4.49 ± 0.16^de^
	4	48	53.06 ± 2.87^cd^	10.41 ± 0.33^cd^
		72	147.75 ± 33.48^b^	21.44 ± 4.72^b^
		96	-26.02 ± 1.20^ef^	-4.52 ± 0.22^fgh^
	6	48	-53.15 ± 3.71^fg^	-7.68 ± 0.52^ghi^
		72	-89.35 ± 2.75^gh^	-9.66 ± 0.30^hi^
		96	47.06 ± 9.58^cd^	7.62 ± 1.62^cde^
Control	-	48	10.80 ± 0.99^de^	1.00 ± 0.09^ef^
		72	11.75 ± 0.35^de^	1.08 ± 0.03^ef^
		96	16.05 ± 0.21^de^	1.50 ± 0.02^ef^

*MeJA* methyl jasmonate; ΔDW: mg [dry weight_after elicitation_ – dry weight_before elicitation_]; GI: % Growth index [(Δdry weight)/dry weight_before elicitation_]. Data presented as mean ± SD of three replicates. Values followed by the same letters in each column are not significantly different according to Duncan’s test (*P* ≤ 0.05)

### Effect of elicitation on TPC and TFC

Depending of concentration and exposure time, elicitation significantly increased or decreased the TPC and TFC of elicited hairy roots cultures (Table 3). The values of TPC and TFC were significantly higher in the 72-h treatments with MeJA (100 and 200 µM), *P. indica* cell extract (4% v/v), and 96-h treatments with *P. indica* culture filtrate (TPC: 4 & 6% v/v, TFC: 2 & 6% v/v) compared to control (*p* < 0.05). Based on these results, the highest yield of TPC was 78.26 mg GAE/g DW and it was found in hairy root cultures elicited with 6% v/v *P. indica* culture filtrate for 96 h. The maximum levels of TFC were obtained from hairy root cultures elicited with 100 and 200 µM MeJA after 72 h which were 3.68 mg GAE/g DW and 3.93 mg QE/g DW, respectively as compared to control (2.66 mg QE/g DW). Compared to the control, the lowest amount of TPC (39% reduction) and TFC (36% reduction) in hairy root cultures was observed after 96 h treatment with 4% v/v cell extract (Table 3).

**Table 3:** Effect of elicitation on total phenolic/flavonoid compounds and antioxidant activity in *F. carica* cv. Siah hairy root cultures

Elicitor	Concen.	Time (h)	TPC (mg GAE/g DW)	TFC (mg QE/g DW)	DPPH assay (%)	FRAP assay (M/g DW)
MeJA [24]	µM					
	100	48	43.42 ± 5.36^fg^	2.79 ± 0.29^e^	7.34 ± 1.59^no^	55.35 ± 5.38^ghi^
		72	55.95 ± 0.32^cd^	3.68 ± 0.15^ab^	23.89 ± 0.94^e^	65.44 ± 7.02^cdef^
		96	28.42 ± 0.73^jkl^	2.80 ± 0.08^e^	6.45 ± 0.61^o^	59.32 ± 4.91^efgh^
	200	48	33.25 ± 0.89^ij^	3.11 ± 0.03^d^	10.32 ± 0.56^lm^	65.61 ± 2.57^cdef^
		72	54.52 ± 0.41^de^	3.93 ± 0.25^a^	7.54 ± 0.19^no^	68.25 ± 3.98^cd^
		96	19.57 ± 0.73^no^	1.89 ± 0.08^kl^	10.96 ± 0.48^klm^	66.43 ± 1.41^cde^
	300	48	26.46 ± 1.71^lm^	1.13 ± 0.25^n^	14.79 ± 2.11^ghi^	53.20 ± 3.74^hij^
		72	27.84 ± 1.38^klm^	1.62 ± 0.1^lm^	16.45 ± 2.11^fg^	60.31 ± 3.51^defgh^
		96	16.98 ± 2.93^op^	1.52 ± 0.1^1m^	15.65 ± 0.23^gh^	62.79 ± 2.81^cdefg^
*P. indica* culture filtrate	% v/v					
	2	48	29.8 ± 2.15^jkl^	2.22 ± 0.15^hij^	13.63 ± 1.65^hij^	59.82 ± 1.40^defgh^
		72	35.3 ± 1.10^hi^	2.55 ± 0.22^efg^	6.68 ± 0.1^o^	64.3 ± 9.76^cdef^
		96	36.98 ± 1.30^hi^	3.32 ± 0.20^cd^	18.36 ± 0.3^f^	61.14 ± 2.97^defgh^
	4	48	44.7 ± 8.04^f^	2.82 ± 0.27^e^	23.50 ± 0.27^e^	66.8 ± 2.31^cde^
		72	26.23 ± 1.66^lm^	2.46 ± 0.1^fgh^	36.36 ± 2.7^b^	60.48 ± 1.00^defgh^
		96	67.3 ± 6.33^b^	2.34 ± 0.23^ghij^	11.7 ± 0.17^jkl^	71.4 ± 2.32^c^
	6	48	50.2 ± 2.64^e^	2.11 ± 0.17^jk^	27.83 ± 0.63^d^	66.43 ± 5.29^cde^
		72	55.4 ± 4.77^cd^	2.16 ± 0.16^ijk^	37.4 ± 1.7^ab^	57.00 ± 0.50^fghi^
		96	78.26 ± 5.52^a^	3.54 ± 0.20^bc^	24.2 ± 0.14^e^	79.67 ± 4.30^b^
*P. indica* cell extract	% v/v					
	2	48	28.02 ± 0.23^jkl^	1.45 ± 0.27^m^	15.81 ± 0.56^gh^	61.5 ± 13.1^defgh^
		72	14.62 ± 2.47^pq^	1.10 ± 0.20^n^	39.24 ± 0.90^a^	57.00 ± 15.30^fghi^
		96	23.87 ± 1.95^mn^	2.29 ± 0.15^ghij^	14.29 ± 0.56^ghi^	67.93 ± 1.83^cde^
	4	48	27.70 ± 2.40^klm^	1.40 ± 0.10^m^	3.74 ± 0.50^p^	59.98 ± 2.00^defgh^
		72	60.03 ± 0.86^c^	3.37 ± 0.06^cd^	30.97 ± 4.43^c^	94.1 ± 1.82^a^
		96	11.50 ± 0.10^q^	0.96 ± 0.30^n^	18.00 ± 1.10^f^	49.7 ± 1.64^ij^
	6	48	32.04 ± 0.70^ijk^	1.87 ± 0.10^kl^	18.30 ± 0.14^f^	65.60 ± 1.82^cdef^
		72	35.02 ± 0.12^hi^	1.59 ± 0.15^m^	9.13 ± 0.27^mn^	68.42 ± 4.30^cd^
		96	39.50 ± 0.11^gh^	2.45 ± 0.02^fghi^	27.53 ± 1.00^d^	70.74 ± 0.60^c^
Control	-	48	29.66 ± 2.23^jkl^	2.68 ± 0.16^ef^	13.25 ± 0.92^ijk^	45.97 ± 1.07^j^
		72	29.62 ± 0.9^jkl^	2.66 ± 0.09^ef^	13.13 ± 0.52^ijk^	46.24 ± 0.66^j^
		96	29.58 ± 1.09^jkl^	2.64 ± 0.07^ef^	12.95 ± 0.56^ijk^	45.5 ± 0.61^j^

*MeJA* methyl jasmonate; TPC: total phenolic content; TFC: total flavonoid content; DPPH: 2, 2-diphenyl-1-picrylhydrazyl radical scavenging capacity; FRAP: ferric reducing antioxidant power. Values represent the means of three replicates ± SD. Values followed by the same letters in each column are not significantly different according to Duncan’s test (*P* ≤ 0.05)

### Effect of elicitation on antioxidant activity

The antioxidant activity of *F. carica* hairy root cultures was evaluated based on two DPPH and FRAP methods. The presence of fungal elicitors in hairy root cultures of *F. carica* than MeJA elicitor caused greater DPPH and FRAP antioxidant activities compared to the control (*p* < 0.05) (Table 3). The highest DPPH antioxidant activity was found in hairy root cultures that were elicited with 2% cell extrac for 72 h (39.24%) followed by 6% and 4% culture filtrate for 72 h (37.4% and 36.36%, respectivly) compared to the control (13.13%). The lowest DPPH antioxidant activity was detected in the hairy roots elicited with 4% cell extract for 48 h (3.74%) (Table 3).

When hairy roots were elicited with MeJA and fungal elicitors, except in two cases (300 µM MeJA for 48 h & 4% v/v fungal cell extract for 96 h), the antioxidant activity of FRAP was significantly increased compared to control hairy root cultures (Table 3). Hairy root cultures elicited with 4% v/v cell extract showed the highest FRAP antioxidant activity (94.1 M/g DW) which was 2-fold higher than that of the control (46.24 M/g DW) (Table 3).

### HPLC analysis of phenolic acids and flavonoids content after elicitation

The amount of gallic, caffeic, chlorogenic, coumaric, rosmarinic and cinnamic phenolic acids, and also rutin, quercetin, and apigenin flavonoids in elicited and non-elicited hairy roots of *F.carica* was quantified by HPLC (Table 4). The level of gallic acid in *F. carica* hairy root culture was considerably raised (48.56-fold) after 96 h exposure to culture filtrate (6% v/v) compared to the control (Table 4). The presence of MeJA in the culture of hairy roots except for a period of 96 h caused a significant increase in gallic acid, and the most increase (5.89 times) was observed in 72-h treatment with a 100 µM (Table 4). The maximum amount of caffeic acid (9.04-fold than control) was detected in cell extract (4% v/v, 72 h) elicited hairy root cultures of *F. carica* (Table 4). The highest yields of chlorogenic acid, coumaric acid, rosmarinic acid and cinnamic acid were obtained in the hairy root cultures treated with MeJA, in dose/time-dependent, as compared to control (Table 4). The MeJA treatments resulting in the highest increase were 100 µM -72 h for chlorogenic acid (10.48-folds), 300 µM-96 h for coumaric acid (8.67-folds), 200 µM- 48 h for rosmarinic acid (12.27-folds), and 300 µM-72 h for cinnamic acid (18.99-folds). As shown in Table 4, the highest levels of rutin, quercetin, and apigenin were found from 6% v/v culture filtrate-treatments, which induced enhancement about 3.34- fold in rutin and 51.96- fold in apigenin after 72 h, and 6.91- fold in quercetin after 96 h.

**Table 4:** Effect of elicitation on the content of phenolic acids and flavonoid compounds in HPLC analysis of *F. carica* cv. Siah hairy root cultures

Elicitor	Concen.	time (h)	Phenolic acid µg/g					Flavonoid (µg/g)			
			Gal	Caf	Chl	Cou	Ros	Cin	Rut	Que	Api
	(µM)										
MeJA [24]	100	48	125.04^j^	177.56^e^	220.8^r^	54.62^o^	79.7^j^	6.86^kl^	5.49^qr^	42.54^s^	17.2^s^
		72	709.7^b^	45.9^s^	6499.93^a^	40.83^q^	137.6^g^	20.87^d^	34.42^b^	22.46^v^	31.58^o^
		96	43.89^v^	48.54^r^	73.41^x^	78.08^n^	30.6^q^	6.95^jk^	13.69^jk^	82.97^l^	11.47^t^
	200	48	122.2^k^	94.96^j^	285.05^p^	318.26^b^	1842.8^a^	9.81^g^	29.53^c^	20.87^w^	47.1^m^
		72	159.01^f^	117.63^h^	36.93^z^	81.75^m^	65.1^l^	5.86^l^	11.19^lm^	31.15^u^	70.7^i^
		96	89.07^r^	60.29^o^	160.91^t^	144.47^g^	243.2^c^	4.33^mn^	10.43^lmn^	33.31^t^	7.9^u^
	300	48	145.5^h^	76.4^m^	285.1^p^	114.16^j^	73.2^k^	2.55^pq^	11.32^l^	120.4^g^	30.46^p^
		72	171.85^e^	221.59^c^	846.35^k^	251.51^c^	482.5^b^	66.09^a^	27.8^d^	199.6^d^	120.72^f^
		96	99.32^q^	119.61^g^	209.16^s^	478.53^a^	57.3^m^	8.09^hi^	25.44^e^	30.91^u^	26.15^q^
*P. indica* culture filtrate	(% v/v)										
	2	48	116.5^m^	61.9^n^	7.07^z^	88.7^l^	-	6.6^kl^	16^h^	45.8^r^	54^k^
		72	145.3^h^	45.6^s^	68.8^y^	52.8^p^	17.7^s^	1.7^rs^	7^pq^	114^i^	49.9^l^
		96	116.6^m^	89.2^k^	79.47^w^	128^h^	26.4^r^	14^f^	11.8^l^	119^h^	76.9^h^
	4	48	126.6^i^	42.5^t^	124.5^v^	103^k^	-	7.3^jk^	13.4^k^	69.7^o^	30^p^
		72	159.8^f^	13.9^x^	258.4^q^	88.6^l^	93.2^i^	3.3^opq^	14.7^ij^	54.8^p^	32.2^o^
		96	193.1^d^	33.8^u^	316.4^o^	117^i^	109^h^	4.1^mno^	5.07^r^	54.8^p^	38.2^n^
	6	48	51.21^u^	106^i^	675.2^l^	145^g^	-	8^ij^	7.09^p^	54^p^	38.2^n^
		72	74.83^s^	17.3^v^	1001.6^h^	25.3^r^	-	19^e^	43.5^a^	73.5^n^	1167^a^
		96	5848^a^	217^d^	1301.5^f^	185^e^	49.1^p^	3.7^mnop^	8.05^p^	341^a^	130^e^
*P. indica* cell extract	(% v/v)										
	2	48	110.2^n^	176.5^f^	996.82^i^	19.7^t^	186^e^	42^b^	10.3^mno^	233^b^	77.4^h^
		72	156.7^g^	300.5^b^	1642.3^c^	151^f^	55^n^	4.7^m^	19.1^g^	115^i^	109^g^
		96	16.66^x^	16.06^w^	147.34^u^	3.7^w^	-	1.7^rs^	3.21^s^	33.9^t^	5.19^v^
	4	48	98.82^q^	48.79^r^	1145^g^	79.2^n^	50^p^	1.3^s^	9.41^o^	154^f^	162^d^
		72	318.6^c^	508.2^a^	2613.3^b^	197^d^	80^j^	2.5^pqr^	24.8^f^	162^e^	295^b^
		96	106.3^p^	119.6^g^	1497.1^d^	24.4^rs^	-	8^ij^	15.6^hi^	93^j^	26^q^
	6	48	108.8^o^	82.7^l^	988.97^j^	16.9^u^	-	9.3^h^	10^no^	227^c^	65.6^j^
		72	19.1^w^	50.58^q^	354.84^n^	14.1^v^	191^d^	29^c^	11.15^lm^	78^m^	191^c^
		96	56.8^t^	49.64^qr^	1424.1^e^	23.6^s^	53^o^	4.2^mno^	11.72^l^	89.3^k^	30.4^p^
Control	-	48	120.14^l^	56.17^p^	620.21^m^	55.30^o^	150.26^f^	3.47^nop^	13.07^k^	49.29^q^	22.53^r^
		72	120.30^l^	56.09^p^	620.32^m^	54.96^o^	150.31^f^	3.46^nop^	13.05^k^	49.32^q^	22.24^r^
		96	120.82^l^	56.43^p^	619.98^m^	54.87^o^	150.03^f^	3.51^nop^	12.94^k^	49.41^q^	22.61^r^

*MeJA* methyl jasmonate; Gal: gallic acid; Caf: caffeic acid; Chl: chlorogenic acid; Rut: rutin; Cou: coumaric acid; Ros: rosmarinic acid; Que: quercetin; Cin: cinnamic acid; Api: apigenin; -, not detected. Values represent the means of three replicates. Values followed by the same letters in each column are not significantly different according to Duncan’s test (*P* ≤ 0.05)

### Expression level of genes affected by elicitation

**Fig. 5:**
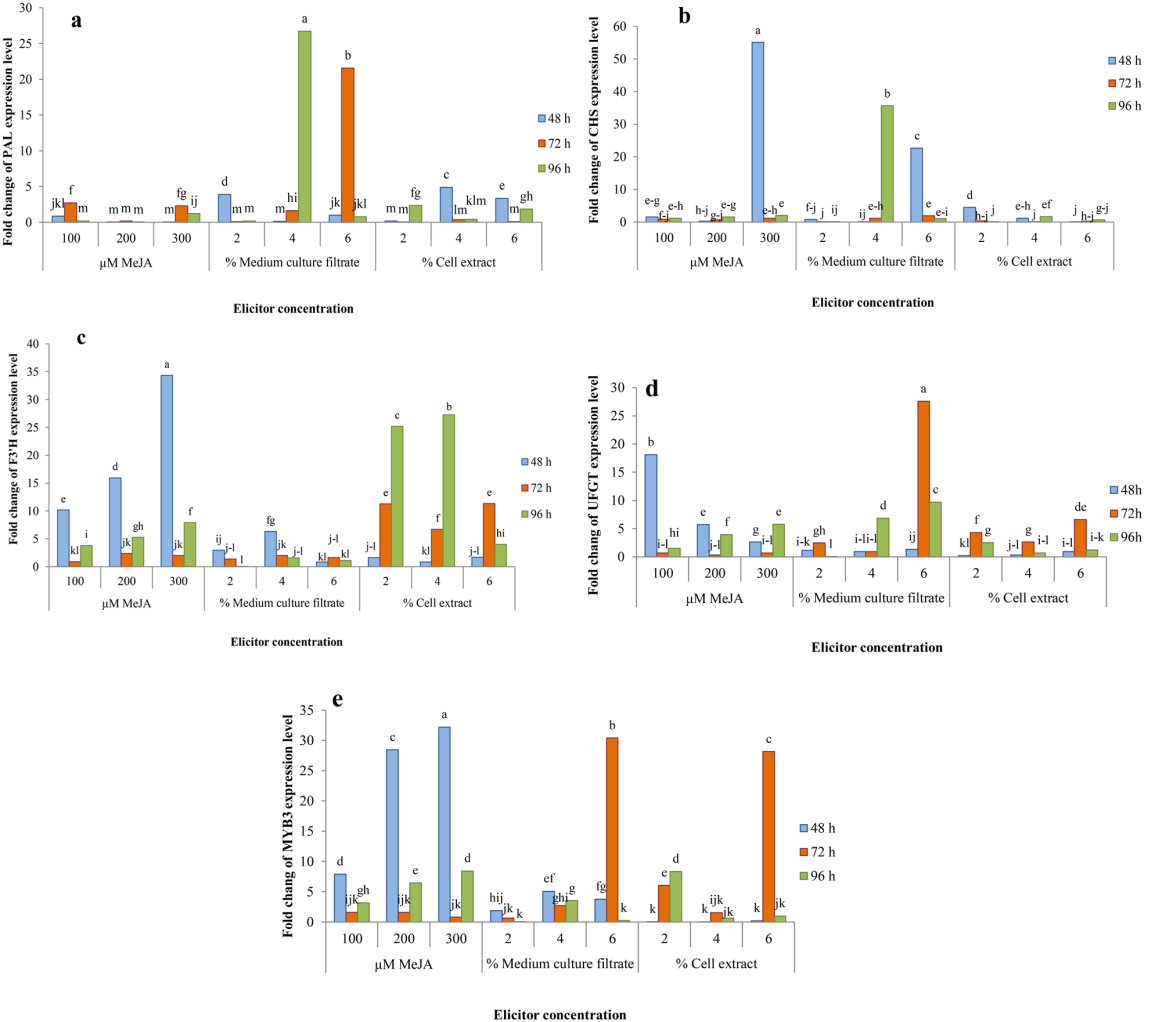
RT-qPCR analysis of different genes from the phenolic/flavonoid biosynthetic pathway, **a**: *PAL*; **b**: *CHS*; **c**: *F3’H*; **d**: *UFGT*; **e**: *MYB3* gene expression in *F. carica* cv. Siah hairy root cultures after elicitation treatments. Values represent the means of three replicates ± SD. Values followed by the same letters in each column are not significantly different according to Duncan’s test (*P* ≤ 0.05)

**Fig. 6:**
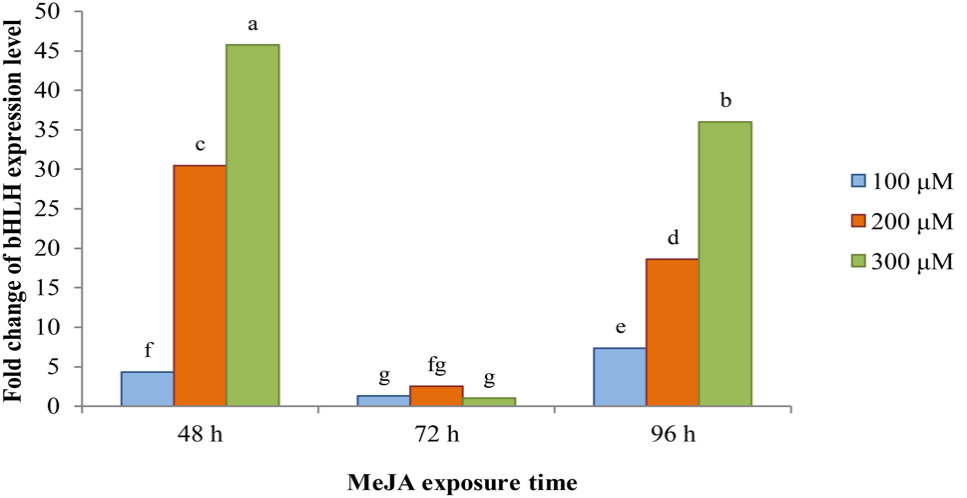
RT-qPCR analysis of bHLH in *F. carica* cv. Siah hairy root cultures after MeJA elicitation treatments. Values represent the means of three replicates ± SD. Values followed by the same letters in each column are not significantly different according to Duncan’s test (*P* ≤ 0.05)

The effect of MeJA and fungal elicitors on the expression levels of *PAL*, *CHS*, *F3’H*, *UFGT*, *MYB3* and *bHLH* genes was analyzed by real-time quantitive PCR in elicited *F. carica* hairy root cultures and compared with the control. **PAL** The highest expression level of *PAL* gene was observed post treatment with *P. indica* culture filtrate elicitor with concentrations of 4% (26.72 fold change) followed by 6% (21.56 fold change) after 96 and 72 h, respectively (Fig. 5, a). In the exposure of *P. indica* cell extract elicitor, the *PAL* gene was expressed higher in concentrations of 4% v/v (4.9 fold change) and 6% v/v (3.36 fold change) at 48 h post treatment. In the presence of MeJA elicitor, *PAL* gene expression was higher in 72-h treatments with concentrations of 100 and 300 µM (2.7 and 2.31 fold change, respectively) compared to other cases (Fig. 5, a). **CHS** The highest increase in the expression of *CHS* gene was related to the MeJA elicitor (55.1- fold change at 300 µM for 48 h) and next peaks was related to the *P. indica* culture filtrate elicitor (35.63- fold change at 4% v/v for 96 h, and 22.71-fold change at 6% v/v for 48 h) compared to control(Fig. 5, b). **F3’H** The highest expression peak of *F3’H* gene (34.33 fold change) was obtained in the *F. carica* hairy root cultures elicited with 300 µM MeJA at 48 h (Fig. 5, c).The results showed that in 48-h treatments with MeJA, the expression level of *F3’H* gene gradually increased with the increase in the elicitor concentration, so that it reached its highest level at the concentration of 300 µM (Fig. 5, c). In the presence of *P. indica* cell extract elicitor, unlike *P. indica* culture filtrate elicitor, at 4% and 2% concentrations, *F3’H* gene expression gradually increased with time and peaked at 96 h (27.28 and 25.2 fold change, respectively) compared to control (Fig. 5, c). **UFGT** The level of *UFGT* expression showed a first peak (27.57 fold change) at 72 h after treatment with 6% medium culture filtrate and second peak (18.13 fold change) at 48 h after treatment with 100 µM MeJA (Fig. 5, d). In exposure to MeJA, 48-h treatments with lower concentrations and 96-h treatments with higher concentrations, and in exposure to *P. indica* culture filtrate and cell extract, 72-h treatments with higher concentrations (exept 4% concentration) were more effective in increasing *UFGT* gene expression in comparison with control (Fig. 5, d). **MYB3** The highest MYB expression level was obtained at 48 h after 300 µM MeJA exposure (32.22 fold change) compared to control (Fig. 5, e). The next high levels of MYB expression were 30.38-fold at 72 h with 6% v/v *P. indica* culture filtrate exposure, and 28.44-fold at 48 h after exposure to 200 µM MeJA or 28.15-fold at 72 h after 6% v/v *P. indica* cell extract treatment (Fig. 5, e). Therefore, in MeJA treatments, higher concentrations at exposure times of 48 h and 96 h, and in fungal treatments, the highest concentration (6% v/v) at exposure time of 72 h caused higher levels of *MYB* expression compared to control (Fig. 5, e). **bHLH** The results showed that MeJA could enhance expression level of bHLH in *F. carica* cv. Siah hairy roots. The 48- and 96-h treatments with higher MeJA concentrations were the leaders in increasing *bHLH* expression, and therefore the lowest increase in expression (1-2.54 fold change) was observed in the 72-h treatments (Fig. 6). In this regard, the maximum level of *bHLH* gene expression belonged to the 48-h treatment (45.73 fold change) and then to the 96-h treatment (36 fold change) with 300 µM concentration of MeJA (Fig. 6).

## Discussion

The current study has proven that elicitors can affect growth index, total phenolic/flavonoid compounds, antioxidant activity, transcriptomic level of some genes/transcription factors involved in phenolic/flavonoid biosynthesis pathway, and concentration of some phenolic acids and flavonoids in elicited hairy root cultures of *F. carica* var. Siah. It has been proposed that one of the ways to increase the synthesis of secondary metabolites in hairy root cultures is the use of elicitor in the culture medium [35]. Elicitation can also be considered an optimal controlled platform to investigate the stress mechanism and possible defense responses of plant cells [36]. Elicitors could induce stress conditions in in vitro plant cultures, as a result lead to the activation of defense mechanisms, subsequently increase the biosynthesis and accumulation of secondary metabolites and even the production of novel metabolites in cultures [37, 38]. The elicitors used in this work were MeJA (wellknown abiotic elicitor) and fungal elicitors, (culture filtrate and cell extract of *P. indica* as biotic elicitors). We found that elicitation of *F. carica* hairy roots with MeJA caused a general decrease in the growth index of hairy roots, but in fungal elicitation, hairy roots growth index can increase or decrease depending on concentration and time of treatment. Biomass inhibition has been reported as one of the effects of high concentrations of elicitors [39]. Jasmonates including jasmonic acid (JA) or methyl jasmonate (MeJA) are phytohormons that as signal transduction elicitor, play a master role in activating the defense responses against pathogens and herbivores, thereby shift metabolism from general to secondary metabolism [40]. According to reports, the activation of defense responses in plant often results in growth inhibition [40]. *P. indica* resembles common arbuscular mycorrhizae that can be easily cultivated under in vitro axenic conditions and used as elicitor [41]. It is reported that elicitation of in vitro plant cultures of *Artemisia annua* with *P. indica* culture filtrate improved the overall growth including root and shoot length, fresh and dry weight [42]. The positive effects of *P. indica* elicitor on in vitro plant growth parameters may be related to its potential in the synthesis of phytohormones, especially auxin [42]. Similar to MeJA, *P. indica* elicitors can also activate the defense mechanism in plant cultures and improve the synthesis of secondary metabolites depending on the treatment concentration and incubation time [43–45].

The induction of plant defenses after elicitation is usually associated with changes in the content of a variety of defensive secondary metabolites, including terpenoids, flavonoids, alkaloids, polyphenols and phenylpropanoids by inducing modifications in related defense genes expression [46]. Fungal compounds mimic stress conditions as an elicitor in plant cell cultures [47]. The present study has confirmed that MeJA and fungal elicitors both could induce different amounts of phenolic/flavonoid compounds depending on the concentration and time of treatment. It is clear that secondary metabolites accumulation can be the result of changes in the expression level of genes [48]. Therefore, in this study, to better understand the changes in the content of phenolic/flavonoid compounds by elicitation, key biosynthetic genes including *PAL*, *CHS*, *UFGT* and *F3’H* genes and MYB3, bHLH transcription factors were analyzed using qRT-PCR. In our previous study, although due to the lack of expression of *F3’H* and *BHLH* genes in the control sample, we failed to analyze the expression of these genes after elicitation, but we found that fungal elicitation was able to up-regulate *PAL*, *CHS*, and *UFGT* and *MBYB3* genes in hairy root cultures of *F. carica* cv. Sabz [8]. Based on this study, in *F. carica* cv. Siah hairy root cultures, the up-regulation of *CHS*, *F3’H*, *MYB3* and *bHLH* genes was induced by MeJA elicitation, while significant highest expression level of *PAL* and *UFGT* genes was induced by fungal elicitation. In our study, the expression level of *PAL* gene showed positive significant correlation with TPC (*r* = 0.383, *p* < 0.05), TFC (*r* = 0.651, *p* < 0.0001), and gallic acid (*r* = 514, *p* < 0.01). The content of apigenin was significant positively correlated with the expression level of *PAL*, *UFGT* and *MYB3* genes. The correlative positive of different treatments (or different elicitors) on antioxidant activity were as : positive significants between DPPH and PAL expression level (*r* = 0.779, *p* < 0.01), gallic acid (*r* = 0.767, *p* < 0.01), chlorogenic acid (*r* = 0.787, *p* < 0.01), and rutin (*r* = 685, *p* < 0.05) after MeJA elicitation; positive significants between DPPH and MYB expression level (*r* = 604, *p* < 0.05), and between FRAP and gallic acid (*r* = 0.578, *p* < 0.05), coumaric acid (*r* = 0.774, *p* < 0.01), and quercetin (*r* = 0.629, *p* < 0.05) after *P. indica* culture filtrate elicitation; positive significants between DPPH and caffeic acid (*r* = 0.705, *p* < 0.05), clorogenic acid (*r* = 0.693, *p* < 0.05), coumaric acid (*r* = 0.595, *p* < 0.05), and rutin (*r* = 0.651, *p* < 0.05) after *P. indica* cell extract elicitation.

It has been reported that the MeJA elicitation improved the content of rosmarinic acid, caffeic acid, chlorogenic acid and cinnamic acid by increasing the relative expression level of *PAL*, *C4H*, *4CL* and *HPPR* genes in *Mentha spicata* hairy root cultures [49]. Increase in TPC, TFC, rosmarinic acid accumulation and transcrional levels of *HPPR*, *PAL*, *C4H*, *4CL1*, *4CL2*, and *CYP98A101*genes in hairy root cultures of *Prunella vulgaris* was induced by methyl jasmonate [21]. In the hairy root cultures of *Centella asiatica* (L.) Urban, 100 µM MeJA enhanced asiaticoside production through increased CabAS (*C. asiatica* putative β-amyrin synthase) gene expression 12 h after elicitation [20]. Fungal elicitors (mycelia extracts of *Fusarium oxysporum* f.sp. lini: non pathogenic, *Phoma exigua*: pathogenic non necrotrophic and *Botrytis cinerea*: pathogenic necrotrophic) were able to significantly increase hypericin and pseudohypericin, TPC, TFC, total anthocyanins, total flavanols and enzymatic activity of PAL and chalcone isomerase (CHI) in *Hypericum perforatum* L. cell cultures [47]. After *P. indica* elicitation, defensive compounds flavonoids, echinacoside and acteoside were increased by increasing the activity of PAL and cinnamyl alcohol dehydrogenase (CAD) enzymes in *Scrophularia striata* cell culture [43]. In hairy roots of *Linum album*, *P. indica* elicitors improved content of lignin, lignans, cinnamic acid, ferulic acid, salicylic acid, myricetin, kaempferol, diosmin and the expression level of *PAL*, cinnamyl alcohol dehydrogenase (*CAD*), cinnamoyl-CoA reductase (*CCR*), and pinoresinol‐lariciresinol reductase (*PLR*) genes [50].

It should be noted, however, that although gene expression and production of phenolic/flavonoid compounds were measured at the same times, any correlation between these two events was not necessarily immediately apparent. There is usually a delay between gene expression and the synthesis of secondary metabolites especially in in vitro cultures, as other factors such as post-transcriptional and post-translational regulation may also be involved [48].

## Conclusions

In order to best understanding of the effect of elicitation on secondary metabolism, we have been studying the effect of MeJA and fungal elicitors on gene regulation and secondary metabolites biosynthesis in hairy root cultures of *Ficus carica*. cv. Siah. MeJA and fungal agents, depending on the concentration and time of exposure, were caused changes in the expression level of biosynthetic genes (*PAL*, *CHS*, *F3’H*, *UFGT*, *MYB3*, *bHLH*) and phenol/flavonoid content in *Ficus carica*. cv. Siah hairy root cultures. Fungal elicitors, especially culture filtrate, despite having no negative effect (except in a few cases) on elicited-hairy roots growth, proved to have the scale up potential for industrial applications to enhance antioxidant secondary metabolites in hairy root cultures of *Ficus carica*. cv. Siah. Therefore, this study provided a strong foundation for new insights into the elicitation process and its positive effects on secondary metabolism, which could be useful for metabolic engineering *F. carica* hairy roots.

### Electronic supplementary material

Below is the link to the electronic supplementary material.

Supplementary Material 1

## Data Availability

All data generated or analyzed during this study are included in this paper.

## Abbreviations

ACT: Actin
ANOVA: Analysis Of Variance
bHLH: Basic Helix-Loop-Helix
CHS: Chalcone Synthase
CTAB buffer: Cetyltrimethyl Ammonium Bromide Buffer
DW: Dry Weight
F3’H: Flavonoid 3′-Hydroxylase
FRAP: Ferric Reducing Antioxidant Power
GAE: Gallic Acid
HPLC: High Performance Liquid Chromatography
HR: Hairy Root
MeJA: Methyl Jasmonate
MS medium: Murashige and Skoog medium
PAL: Phenylalanine Ammonia-Lyase
PCR: Polymerase Chain Reaction
QE: quercetin
Real Time RT-PCR: Real-Time Reverse Transcription–Polymerase Chain Reaction
TPC: Total Phenolic Compounds
SD: Standard Deviation
TFC: Total Flavonoid Compounds
UFGT: UDP-Glucose Flavonoid 3-O-Glucosyltransferase

## References

[1] Teruel-Andreu C, Andreu-Coll L, López-Lluch D, Sendra E, Hernández F, Cano-Lamadrid M (2021). *Ficus carica* fruits, By-Products and based products as potential sources of Bioactive compounds: a review. Agronomy.

[2] Saddoud O, Baraket G, Chatti K, Trifi M, Marrakchi M, Salhi-Hannachi A, Mars M (2008). Morphological variability of fig (*Ficus carica* L.) cultivars. Int J Fruit Sci.

[3] Morovati MR, Ghanbari-Movahed M, Barton EM, Farzaei MH, Bishayee A. A systematic review on potential anticancer activities of *Ficus carica* L. with focus on cellular and molecular mechanisms. Phytomedicine 2022:154333. 10.1016/j.phymed.2022.154333. 10.1016/j.phymed.2022.154333 35952577

[4] Li Z, Yang Y, Liu M, Zhang C, Shao J, Hou X, Tian J, Cui Q (2021). A comprehensive review on phytochemistry, bioactivities, toxicity studies, and clinical studies on *Ficus carica* Linn. Leaves. Biomed Pharmacother.

[5] Gurung AB, Ali MA, Lee J, Farah MA, Al-Anazi KM (2021). Molecular docking and dynamics simulation study of bioactive compounds from *Ficus carica* L. with important anticancer drug targets. PLoS ONE.

[6] Oliveira AP, Baptista P, Andrade PB, Martins F, Pereira JA, Silva BM, Valentao P (2012). Characterization of *Ficus carica* L. cultivars by DNA and secondary metabolite analysis: is genetic diversity reflected in the chemical composition?. Food Res Int.

[7] Meziant L, Bachir-bey M. *Ficus carica* L. as a source of Natural Bioactive flavonoids. Fig (Ficus carica): production, Processing, and Properties. Springer; 2023. pp. 417–65.

[8] Amani S, Mohebodini M, Khademvatan S, Jafari M, Kumar V (2021). *Piriformospora indica* based elicitation for overproduction of phenolic compounds by hairy root cultures of *Ficus carica*. J Biotech.

[9] Wawrosch C, Zotchev SB (2021). Production of bioactive plant secondary metabolites through in vitro technologies—status and outlook. Appl Microbiol Biotechnol.

[10] Kowalczyk T, Wieczfinska J, Skała E, Śliwiński T, Sitarek P (2020). Transgenesis as a tool for the efficient production of selected secondary metabolites from plant *in vitro* cultures. Plants.

[11] Supriya R, Kala RG, Thulaseedharan A. Hairy Root Culture: secondary metabolite production in a Biotechnological Perspective. Plant Metabolites: Methods Appl Prospects 2020:89–110. 10.1007/978-981-15-5136-9_5.

[12] Sonkar N, Shukla P, Misra (2023). Plant hairy roots as Biofactory for the production of Industrial metabolites. Plants as Bioreactors Industrial Molecules.

[13] Kaur G, Prakash P, Srivastava R, Verma PC. Enhanced secondary metabolite production in hairy root cultures through biotic and abiotic elicitors. Plant cell Tissue Differ Secondary Metabolites: Fundamentals Appl. 2021;625–60. 10.1007/978-3-030-30185-9_38.

[14] Halder M, Sarkar S, Jha S (2019). Elicitation: a biotechnological tool for enhanced production of secondary metabolites in hairy root cultures. Eng Life Sci.

[15] Meena M, Yadav G, Sonigra P, Nagda A, Mehta T, Swapnil P, Marwal A (2022). Role of elicitors to initiate the induction of systemic resistance in plants to biotic stress. Plant Stress.

[16] Salehi M, Moieni A, Safaie N, Farhadi S (2020). Whole fungal elicitors boost paclitaxel biosynthesis induction in *Corylus avellana* cell culture. PLoS ONE.

[17] Xu W, Jin X, Yang M, Xue S, Luo L, Cao X, Zhang C, Qiao S, Zhang C, Li J (2021). Primary and secondary metabolites produced in *Salvia miltiorrhiza* hairy roots by an endophytic fungal elicitor from *Mucor Fragilis*. Plant Physiol Biochem.

[18] Xu X-d, Liang W-x, Yao L, Paek K-Y, Wang J, Gao W-y (2021). Production of ginsenoside by *Chaetomium* sp. and its effect on enhancing the contents of ginsenosides in *Panax ginseng* adventitious roots. Biochem Eng J.

[19] Ali M, Abbasi BH, Ali GS (2015). Elicitation of antioxidant secondary metabolites with jasmonates and gibberellic acid in cell suspension cultures of *Artemisia absinthium* L. Plant Cell Tissue Organ Cult.

[20] Kim O-T, Bang K-H, Shin Y-S, Lee M-J, Jung S-J, Hyun D-Y, Kim Y-C, Seong N-S, Cha S-W, Hwang B (2007). Enhanced production of asiaticoside from hairy root cultures of *Centella asiatica* (L.) Urban elicited by methyl jasmonate. Plant Cell Rep.

[21] Ru M, Li Y, Guo M, Chen L, Tan Y, Peng L, Liang Z (2022). Increase in rosmarinic acid accumulation and transcriptional responses of synthetic genes in hairy root cultures of *Prunella vulgaris* induced by methyl jasmonate. Plant Cell Tissue Organ Cult.

[22] Xiao Y, Gao S, Di P, Chen J, Chen W, Zhang L (2009). Methyl jasmonate dramatically enhances the accumulation of phenolic acids in *Salvia miltiorrhiza* hairy root cultures. Physiol Plant.

[23] Perassolo M, Cardillo AB, Mugas ML, Montoya SCN, Giulietti AM, Talou JR (2017). Enhancement of anthraquinone production and release by combination of culture medium selection and methyl jasmonate elicitation in hairy root cultures of *Rubia tinctorum*. Ind Crop Prod.

[24] Amani S, Mohebodini M, Khademvatan S, Jafari M (2020). *Agrobacterium rhizogenes* mediated transformation of *Ficus carica* l. for the efficient production of secondary metabolites. J Sci Food Agric.

[25] Lloyd G, McCown B. Commercially-feasible micropropagation of mountain laurel, Kalmia latifolia, by use of shoot-tip culture. In: *International Plant Propagators Society: 1980*. 421–427.

[26] Pirttila AM, Hirsikorpi M, Kamarainen T, Jaakola L, Hohtola A (2001). DNA isolation methods for medicinal and aromatic plants. Plant Mol Biol Rep.

[27] Sambrook J, Russell D. Molecular cloning: A laboratory manual, the third edition. In.: Cold spring harbor laboratory press, cold spring harbor, New York; 2001.

[28] Wang JW, Wu JY. Effective Elicitors and Process Strategies for Enhancement of Secondary Metabolite Production in Hairy Root Cultures. In: *Biotechnology of Hairy Root Systems* Edited by Doran PM. Berlin, Heidelberg: Springer Berlin Heidelberg; 2013: 55–89. 10.1007/10_2013_183. 10.1007/10_2013_183 23467807

[29] Sharan S, Sarin N, Mukhopadhyay K (2019). Elicitor-mediated enhanced accumulation of ursolic acid and eugenol in hairy root cultures of *Ocimum tenuiflorum* L. is age, dose, and duration dependent. South Afr J Bot.

[30] Reid KE, Olsson N, Schlosser J, Peng F, Lund ST (2006). An optimized grapevine RNA isolation procedure and statistical determination of reference genes for real-time RT-PCR during berry development. BMC Plant Biol.

[31] Singleton VL, Rossi JA (1965). Colorimetry of total phenolics with phosphomolybdic-phosphotungstic acid reagents. Am J Enol Vitic.

[32] Bakar MFA, Mohamed M, Rahmat A, Fry J (2009). Phytochemicals and antioxidant activity of different parts of bambangan (*Mangifera Pajang*) and tarap (*Artocarpus odoratissimus*). Food Chem.

[33] Argolo A, Sant’Ana A, Pletsch M, Coelho L (2004). Antioxidant activity of leaf extracts from *Bauhinia monandra*. Bioresour Technol.

[34] Benzie IF, Strain JJ (1996). The ferric reducing ability of plasma (FRAP) as a measure of antioxidant power: the FRAP assay. Anal Biochem.

[35] Wei T, Deng K, Gao Y, Chen L, Song W, Zhang Y, Wang C, Chen C (2020). *SmKSL* overexpression combined with elicitor treatment enhances tanshinone production from *Salvia miltiorrhiza* hairy roots. Biochem Eng J.

[36] Narayani M, Srivastava S (2017). Elicitation: a stimulation of stress in *in vitro* plant cell/tissue cultures for enhancement of secondary metabolite production. Phytochem Rev.

[37] Siddiqui ZH, Mujib A, Mahmooduzzafar, Aslam J, Rehman Hakeem K, Parween T. In vitro production of secondary metabolites using elicitor in Catharanthus roseus: a case study. Crop Improv: New Approaches Mod Techniques. 2013;401–19. 10.1007/978-1-4614-7028-1_14.

[38] Fang L, Sharma AR, Aniemena C, Roedel K, Henry F, Moussou P, Samuga A, Medina-Bolivar F (2022). Elicitation of stilbenes and benzofuran derivatives in hairy root cultures of white mulberry (*Morus alba*). Plants.

[39] An D, Wu C-H, Wang M, Wang M, Chang G-N, Chang X-J, Lian M-L (2022). Methyl jasmonate elicits enhancement of bioactive compound synthesis in adventitious root co-culture of *Echinacea purpurea* and *Echinacea pallida*. Vitro Cell Dev Biol Plant.

[40] Rogowska A, Stpiczyńska M, Pączkowski C, Szakiel A (2022). The influence of exogenous jasmonic acid on the biosynthesis of steroids and triterpenoids in *Calendula officinalis* plants and hairy root culture. Int J Mol Sci.

[41] Baldi A, Srivastava A, Bisaria V (2009). Fungal elicitors for enhanced production of secondary metabolites in plant cell suspension cultures. Symbiotic Fungi.

[42] Baishya D, Deka P, Kalita MC (2015). *In vitro* co-cultivation of *Piriformospora indica* filtrate for improve biomass productivity in *Artemisia annua* (L). Symbiosis.

[43] Shahkarami P, Ahmadian-Chashmi N, Samari E, Safaie N, Sharifi M. *Piriformospora indic*a induces phenylethanoid glycosides production and defense responses in *Scrophularia striata* cell culture. Plant Cell Tissue Organ Cult 2022:1–15. 10.1007/s11240-021-02213-0.

[44] Ahlawat S, Saxena P, Ali A, Khan S, Abdin MZ (2017). Comparative study of withanolide production and the related transcriptional responses of biosynthetic genes in fungi elicited cell suspension culture of *Withania somnifera* in shake flask and bioreactor. Plant Physiol Biochem.

[45] Kumar V, Rajauria G, Sahai V, Bisaria V (2012). Culture filtrate of root endophytic fungus *Piriformospora indica* promotes the growth and lignan production of *Linum album* hairy root cultures. Process Biochem.

[46] Zhao J, Davis LC, Verpoorte R (2005). Elicitor signal transduction leading to production of plant secondary metabolites. Biotechnol Adv.

[47] Gadzovska Simic S, Tusevski O, Maury S, Hano C, Delaunay A, Chabbert B, Lamblin F, Lainé E, Joseph C, Hagège D (2015). Fungal elicitor-mediated enhancement in phenylpropanoid and naphtodianthrone contents of *Hypericum perforatum* L. cell cultures. Plant Cell Tissue Organ Cult.

[48] Alcalde MA, Cusido RM, Moyano E, Palazon J, Bonfill M (2022). Metabolic gene expression and centelloside production in elicited *Centella asiatica* hairy root cultures. Ind Crop Prod.

[49] Yousefian S, Lohrasebi T, Farhadpour M, Haghbeen K (2020). Effect of methyl jasmonate on phenolic acids accumulation and the expression profile of their biosynthesis-related genes in *Mentha spicata* hairy root cultures. Plant Cell Tissue Organ Cult.

[50] Tashackori H, Sharifi M, Chashmi NA, Behmanesh M, Safaie N (2018). *Piriformospora indica* cell wall modulates gene expression and metabolite profile in *Linum album* hairy roots. Planta.

[51] Ikegami H, Habu T, Mori K, Nogata H, Hirata C, Hirashima K, Tashiro K, Kuhara S (2013). De novo sequencing and comparative analysis of expressed sequence tags from gynodioecious fig (*Ficus carica* L.) fruits: caprifig and common fig. Tree Genet Genomes.

[52] Wang Z, Cui Y, Vainstein A, Chen S, Ma H. Regulation of fig (*Ficus carica* L.) fruit color: metabolomic and transcriptomic analyses of the flavonoid biosynthetic pathway. Front Plant Sci. 2017;81990. 10.3389/fpls.2017.01990. 10.3389/fpls.2017.01990 PMC5701927 29209349

