# Supplementary material for: Modifications in gene expression and phenolic compounds content by methyl jasmonate and fungal elicitors in Ficus carica. Cv. Siah hairy root cultures
Source: BMC Plant Biol. 2024 Jun 10;24:520. doi: 10.1186/s12870-024-05178-2 (PMC11163756; doi:10.1186/s12870-024-05178-2)
Supplement: Supplementary file 1 — Supplementary Material 1 [file 12870_2024_5178_MOESM1_ESM.docx]

# Supplemental data

**Figure 1.**


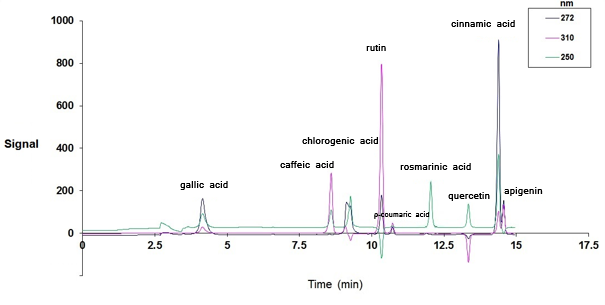


Figure 1. The HPLC chromatogram of a mixture of standards for resolution of nine phenolic compounds, including gallic acid, caffeic acid, chlorogenic acid, rutin, ρ-coumaric acid, rosmarinic acid, quercetin, cinnamic acid, and apigenin at 272 , 250, and 310 nm.

**Original images of blots in Figure 2 and Figure 3:**


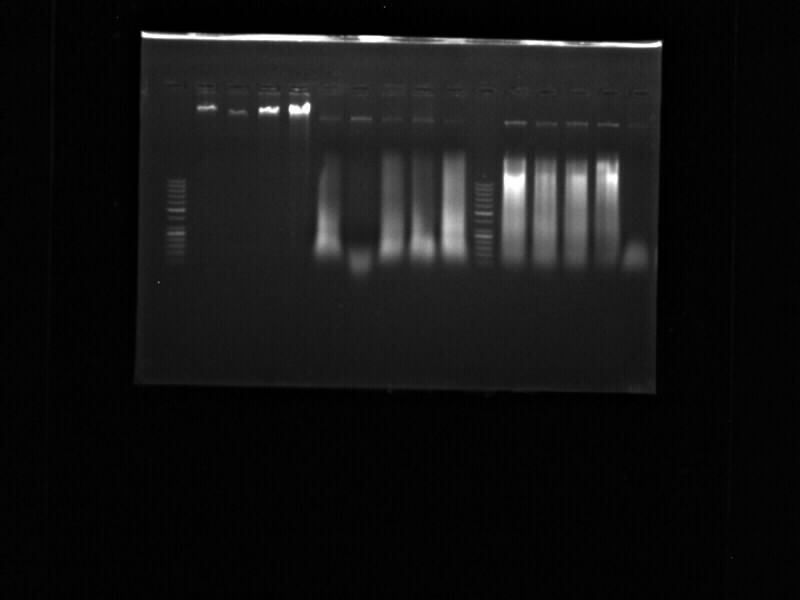


Figure 2 , A: Gel electrophoresis of extracted DNA


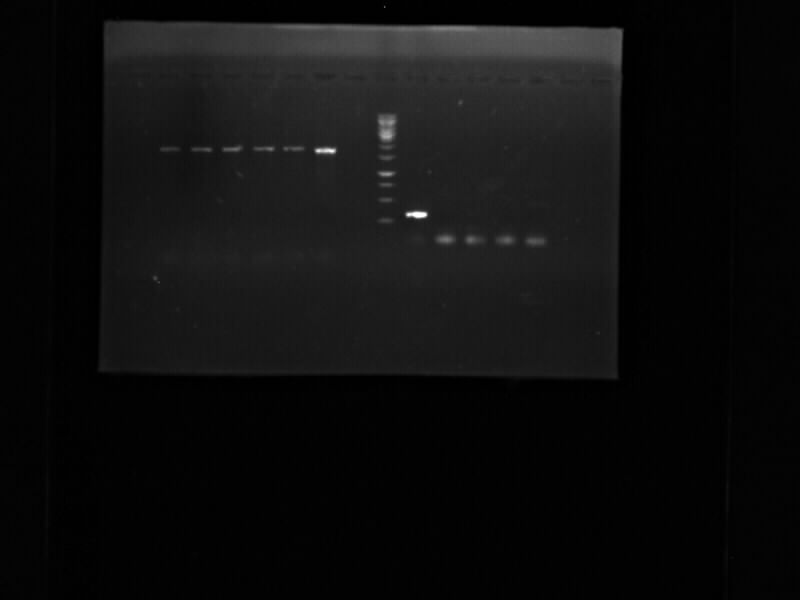


**Figure 2, B1 & B2: B1 (left) PCR amplicons of hairy root lines using rolA-B (1794 bp) primer and B2 (right): PCR amplicons using virD2 (338 bp)**


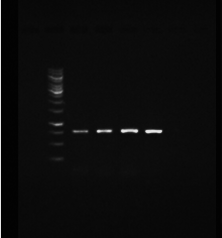


**Figure 2, C: PCR amplicons of three hairy root lines using rolB (780 bp)**


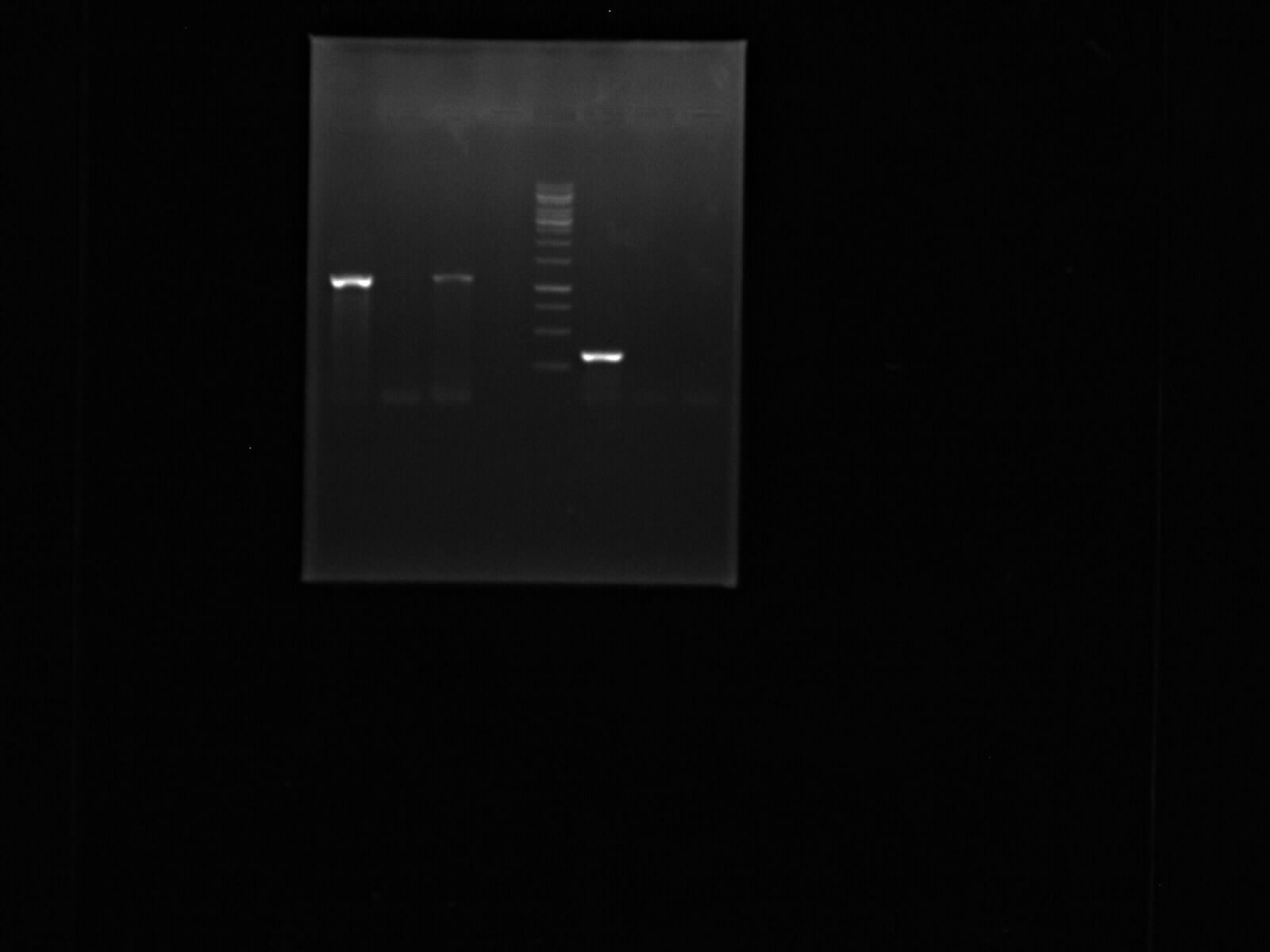


**Figure 2, D: PCR amplicons of L10 hairy root line by rolC-D (1105 bp) and virD2**


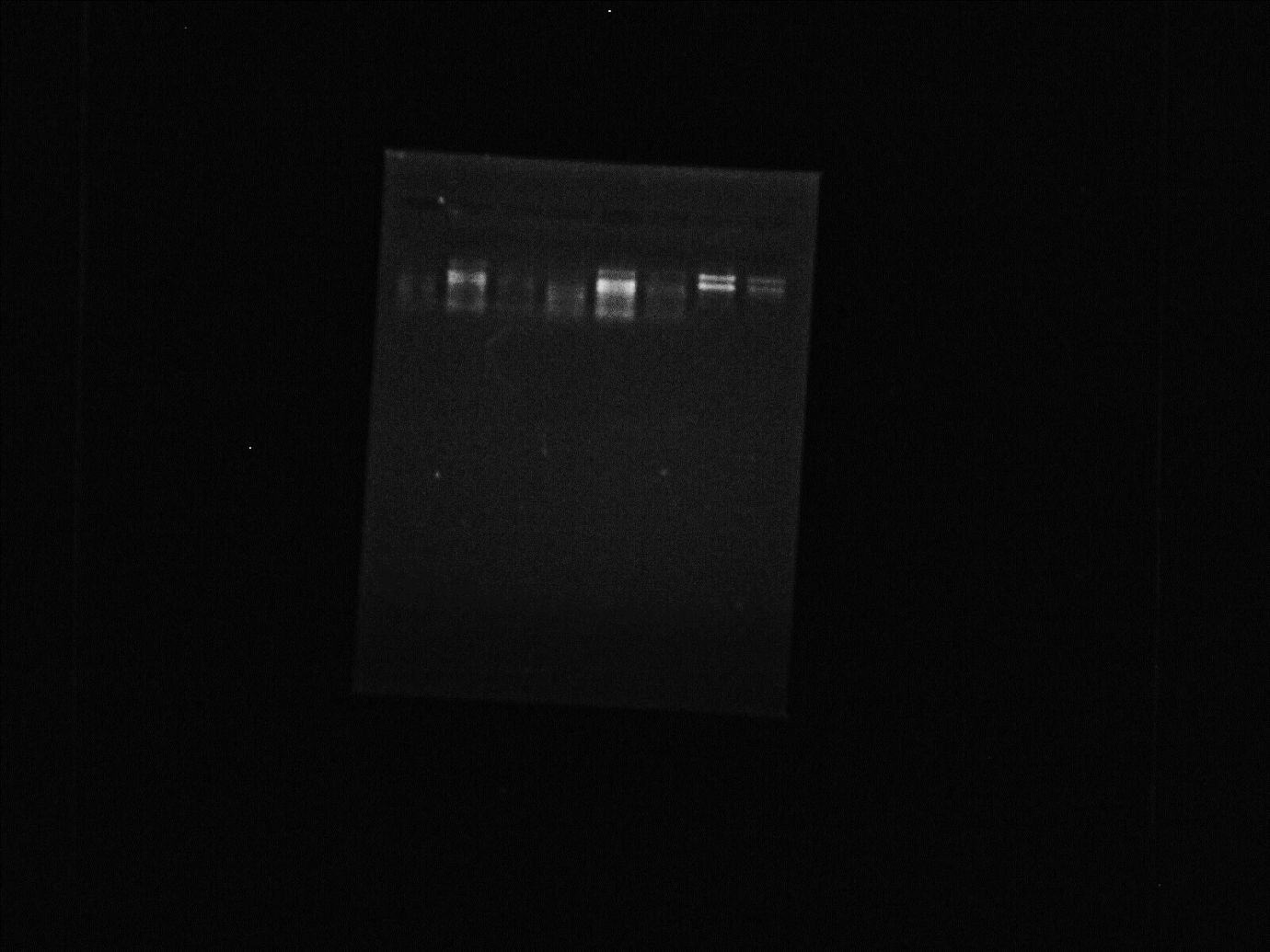


**Figure 2, E: RNA extracted from non-elicited and/or elicited L10 hairy roots (two lase wells).**


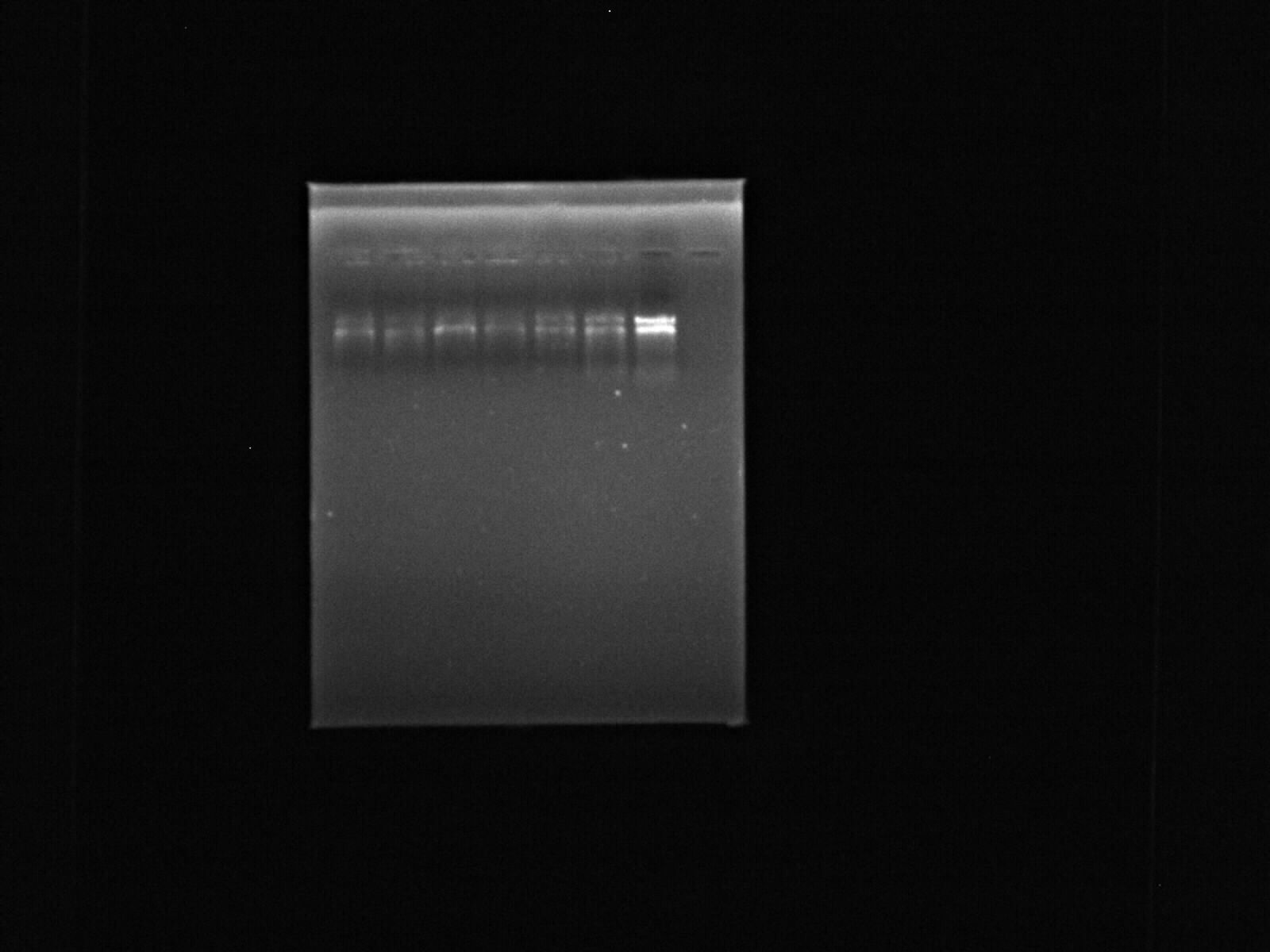


**Figure 2, F: RNA extracted from non-elicited and/or elicited L10 hairy roots.**


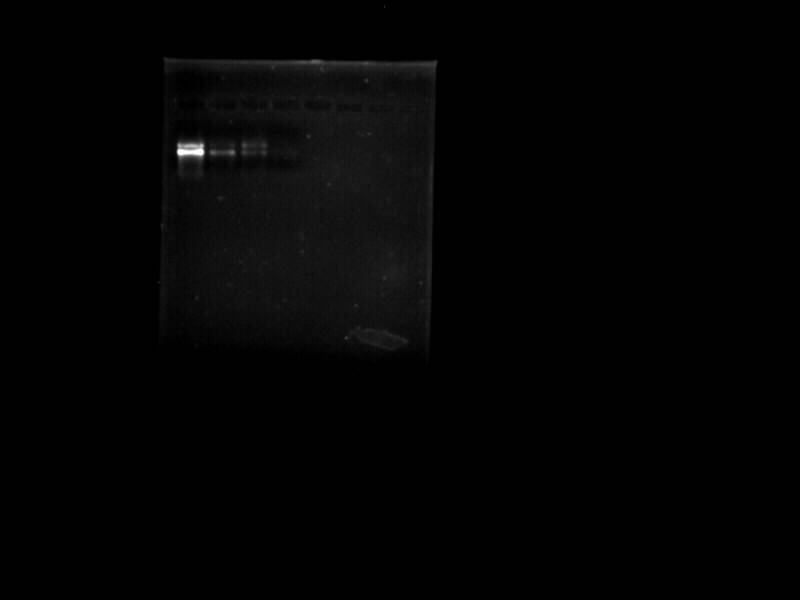


**Figure 2, G: RNA extracted from non-elicited and/or elicited L10 hairy roots.**


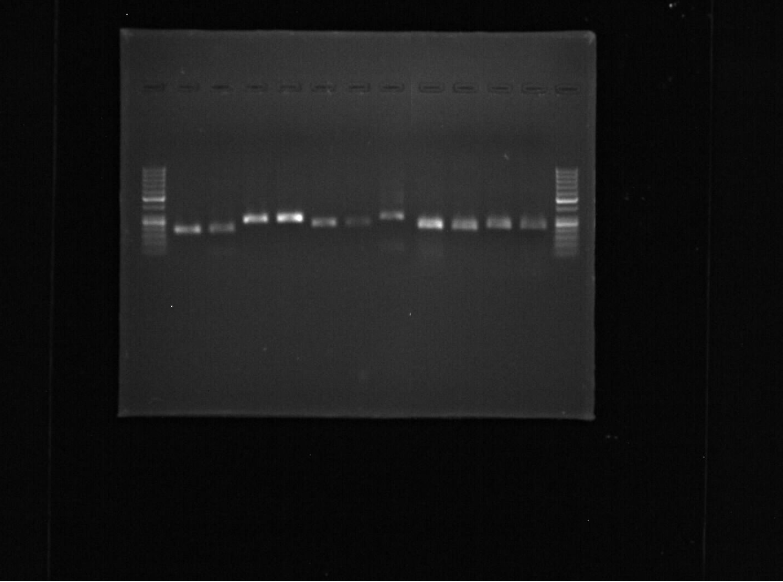


Figure 3, (left part). Gel electrophoresis of RTq-PCR amplicons using specific primers for CHS, PAL, UFGT, bHLH, F3'H and ACT1


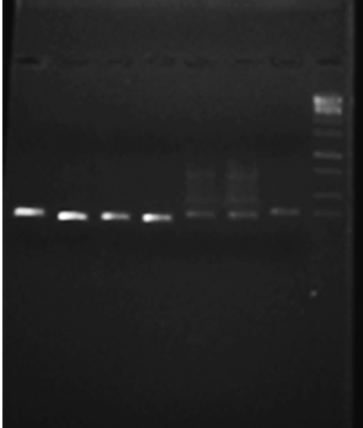


**Figure 3, (right part). Gel electrophoresis of RTq-PCR amplicons using specific primers bHLH, MYB3, F3'H, UFGT and ACT1 (right to left)**
